# Normative Database for All Retinal Layer Thicknesses Using SD-OCT Posterior Pole Algorithm and the Effects of Age, Gender and Axial Lenght

**DOI:** 10.3390/jcm9103317

**Published:** 2020-10-15

**Authors:** Ana Palazon-Cabanes, Begoña Palazon-Cabanes, Elena Rubio-Velazquez, Maria Dolores Lopez-Bernal, Jose Javier Garcia-Medina, Maria Paz Villegas-Perez

**Affiliations:** 1 Department of Ophthalmology, General University Hospital Reina Sofia, 30003 Murcia, Spain; a.palazoncabanes@gmail.com (A.P.-C.); mpville@um.es (M.P.V.-P.); 2 Department of Neurology, University Hospital La Arrixaca, 30005 Murcia, Spain; abega_ct@hotmail.com; 3 Department of Ophthalmology, General University Hospital Morales Meseguer, 30008 Murcia, Spain; elena.rubio@carm.es (E.R.-V.); dolores.lopez11@carm.es (M.D.L.-B.); 4 Department of Ophthalmology and Optometry, University of Murcia, 30120 Murcia, Spain; 5 Ophthalmic Research Unit Santiago Grisolia, 46017 Valencia, Spain; 6 Red Temática de Investigación Cooperativa en Patología Ocular (OFTARED), Instituto de Salud Carlos III, 28029 Madrid, Spain

**Keywords:** macula, retina, normative database, layer, optical coherence tomography, posterior pole algorithm, thickness, 8 × 8, glaucoma, neuro-ophthalmology

## Abstract

Our aim was to provide, for the first time, reference thickness values for the SD-OCT posterior pole algorithm (PPA) available for Spectralis OCT device (Heidelberg Engineering, Heidelberg, Germany) and to analyze the correlations with age, gender and axial length. We recruited 300 eyes of 300 healthy Caucasian subjects between 18 and 84 years. By PPA, composed of 64 (8 × 8) cells, we analyzed the thickness of the following macular layers: retinal nerve fiber layer (RNFL), ganglion cell layer (GCL), inner plexiform layer (IPL), inner nuclear layer (INL), outer plexiform layer (OPL), outer nuclear layer (ONL), retinal pigment epithelium (RPE), inner retina, outer retina and full retina. Mean ± SD, 1st, 5th, 95th percentiles were obtained for each cell at all macular layers. Significant negative correlations were found between age and thickness for most macular layers. The mean thickness of most macular layers was thicker for men than women, except for RNFL, OPL and RPE, with no gender differences. GCL, IPL and INL thicknesses positively correlated with axial length in central cells, and negatively in the cells near the optic disk. The mean RNFL thickness was positively associated with axial length. This is the first normative database for PPA. Age, gender and axial length should be taken into account when interpreting PPA results.

## 1. Introduction

Macula, the most central region of the retina, is a highly specialized area that serves visual acuity, color and shape differentiation and stereopsis, and is responsible for the 18 central degrees of the visual field [1,2].

Many ocular diseases tend to affect the macula. These diseases are usually degenerative diseases that cause neuronal loss and, thus, produce the thickness of the total macula and cause different macular layers to thin [3,4,5]. However, some other diseases also produce increased thickness of macular layers [6]. The early detection of these changes in thickness of different macular layers enables the early diagnosis and treatment of pathologies that frequently cause blindness, such as glaucoma [7] or choroidal neovascularization. It has been demonstrated that neurological disorders, such as multiple sclerosis [8], Alzheimer’s disease [9], Parkinson’s disease [10] and autism [11], as well as psychiatric diseases [12], affect the macula.

Optical coherence tomography (OCT) is a non-invasive reproducible technique that allows in vivo tomographic retina imaging at a resolution approaching that of histological sections [13]. Since it was introduced into the ophthalmology field, this technique has considerably developed, and acquisition speed and image resolution have improved. The most widely used OCT model today is spectral-domain OCT (SD-OCT), which offers a scanning speed of 312.500 axial scans per second and an axial resolution of from 5 to 6 microns [14].

To interpret the images obtained with SD-OCT, normative databases, containing data values from healthy subjects, have been created for each commercial SD-OCT device and/or protocol. At present, most SD-OCT devices contain the normative database only for values of total macular retinal thickness and peripapillary retinal nerve fiber thickness (pRNFL) for the macular cube and optic disk circle protocols, respectively. The SD-OCT software usually permits comparisons of the values acquired with the values in the normative database, and shows the results as color-coded in order to indicate whether the detected thickness values are under the 1st percentile (red), between the 1st and 5th percentiles (yellow), between the 5th and 95th percentiles (green) or over the 95th percentile (white) of the normal reference database.

Researchers worldwide have published the normative database for macular layers thicknesses for various SD-OCT devices, such as Cirrus™ high-definition (HD)-OCT (Carl Zeiss Meditec, Jena, Germany) [15,16] and Spectralis™ SD-OCT (Heidelberg Engineering, Heidelberg, Germany) [17,18], and for the Swept-source (SS)-OCT device (DRI OCT Triton™, Topcon, Tokyo, Japan) [19], among others. These authors have employed different image acquisition protocols and represented data using several algorithms, of which the Early Treatment Diabetic Retinopathy Study (ETDRS) grid is a frequent choice. The aforementioned studies have evaluated different layers, mostly inner retinal layers, and distinct ethnic and age group populations. However, only three research groups [20,21,22] have investigated the normal thickness values of all macular layers by Spectralis™ SD-OCT using the ETDRS map.

Spectralis™ SD-OCT has different exploration protocols for the macula. One of them, the posterior pole algorithm (PPA) permits the automatic segmentation of the individual macular layers. To the best of our knowledge, there is no normative database for the PPA of Spectralis™ SD-OCT and, thus, the protocol cannot compare the resulting values to those of a reference population. The aim of the present study was to develop a normative database for all macular retinal layers for this protocol in a healthy Caucasian population and to analyze the impact of age, gender and axial length on all retinal layer thicknesses.

## 2. Materials and Methods

This observational cross-sectional study included healthy Caucasian volunteers who attended the Department of Ophthalmology at the University General Hospital Reina Sofia in Murcia, Spain, for routine examinations. The participants were chosen proportionally according to gender and age to obtain a wide representative sample of the population [23]. The inclusion criteria were: aged between 18 and 85 years, Caucasian ethnicity, best corrected visual acuity greater than 20/40, normal ophthalmological examination and normal pRNFL thickness. The exclusion criteria were: sphere ≥ 5 diopters and/or cylinder ≥ 2 diopters, adjusted intraocular pressure (IOP) > 21 mmHg, cup to disc ratio > 0.4, recent ocular surgery (in the last 6 months), media opacities leading to a signal strength of the OCT images under 25, any previous or current ocular disease (glaucoma, uveitis, age-related macular degeneration, diabetic retinopathy, etc.) or neuropsychiatric diseases. Only one eye per participant was randomly chosen. Six age groups were distinguished following the normative database of another widely used commercial SD-OCT, Cirrus™ HD-OCT [24]: 18–29, 30–39, 40–49, 50–59, 60–69 and 70–85 years. Each age group included 25 male eyes and 25 female eyes.

The study protocol was approved by the Local Ethics Committee at the University General Hospital Reina Sofia in Murcia, Spain (protocol number 03/19, approval date 29 January 2019) and adhered to the ethical principles stated in the Declaration of Helsinki. Patients were informed about the study and informed consent was obtained from them all before enrollment.

All the participants underwent comprehensive ophthalmologic examination, including visual acuity, slit-lamp biomicroscopy and fundus examination, autorefractometry (NIDEK ARK-710A), pneumatic tonometry (NIDEK NT2000), determination of axial length (AXL), keratometry (IOL Master; Carl Zeiss Meditec) and pachymetry (Specular Microscope EM-3000; Tomey, Aichi, Japan). Furthermore, the SD-OCT images of the macula and optic disk were acquired with Spectralis™ (Heidelberg Engineering, Heidelberg, Germany; version 6.0) using the 8 × 8 PPA and the optic disk circle protocols, respectively. All the OCT examinations were acquired by the same experienced ophthalmologist who visually controlled the head position of every patient to minimize head tilt (A.P.C).

The 8 × 8 PPA scans a macular cube measuring 30° × 25°, centered on the fovea and oriented using a fovea-disk alignment [25]. The protocol represents the results on a macular grid measuring 24° × 24°, that is divided into 64 cells, each measuring 3° × 3°, which are distributed in eight rows and eight columns (8 × 8). The nomenclature of these cells is specular between eyes. Cell numbering proceeds from temporal to nasal, and from inferior to superior. Thus cell 1.1 is the most infero-temporal cell and cell 8.8 is the most supero-nasal cell in both the right and left eyes. Because of the specular nomenclature, the left eye data are shown as a right eye format. In this study, the four most central cells are called foveal cells (4.4, 4.5, 5.4, 5.5), the eight cells immediately external to foveal cells are called parafoveal cells (3.4, 3.5, 4.3, 4.6, 5.3, 5.6, 6.4, 6.5), the 12 cells immediately external to parafoveal cells are called perifoveal cells (2.4, 2.5, 3.3, 3.6, 4.2, 4.7, 5.2, 5.7, 6.3, 6.6, 7.4, 7.5) and the most external cells are called peripheral cells. Using the automatic segmentation tool of this protocol, for each cell we obtained the thickness of the following macular layers: retinal nerve fiber layer (RNFL; from internal limiting membrane (ILM) to RNFL), ganglion cell layer (GCL; from RNFL to GCL), inner plexiform layer (IPL; from GCL to IPL), inner nuclear layer (INL; from IPL to INL), outer plexiform layer (OPL; from ONL to OPL), outer nuclear layer (ONL; from OPL to external limiting membrane) and retinal pigment epithelium (RPE; from upper RPE to Bruch’s membrane). We also obtained the joint automatic segmentation from RNFL to ONL or inner retina (INNER), photoreceptors and the RPE or outer retina (OUTER) and all the retinal layers or total retina (RETINA). The borders considered for the pRNFL segmentation were from ILM and to GCL. All the scans were inspected by the same ophthalmologist (A.P.C) to detect segmentation errors and other artifacts, such as misalignments, decentration or motion artifacts. In these cases, examinations were excluded. Only the high-quality scans with signal strength intensity over 25 were included. No manual adjustments were made.

### Statistical Analysis

We performed all the statistical analyses using the SPSS software (version 26.0; SPSS Inc, Chicago, IL, USA). We tested that all the thickness values and age and AXL variables were normally distributed by the Kolmogorov–Smirnov test. We expressed the quantitative data as mean and standard deviation. We calculated the 1st, 5th and 95th percentiles among same-age subjects in the 64 cells of all the macular layers. We compared macular layers thicknesses between gender by the Student’s *t*-test for the independent samples. We ran the Pearson correlation test to investigate the effects of age and AXL on the thickness parameters. A *p*-value < 0.05 was considered to be statistically significant.

## 3. Results

Three hundred eyes of 300 subjects were included in this study, 150 belonging to men (50%) and 150 to women (50%). The participants’ mean age was 49.78 ± 17.41 years (range 18 to 84). This study included 152 right eyes (51%) and 147 left eyes (49%). The mean AXL was 23.64 ± 0.90 mm (range 21.25 to 25.95). The mean keratometry was 43.25 ± 1.40 diopters for the flattest meridian and 44.14 ± 1.45 diopters for the steepest meridian. The mean IOP was 15.60 ± 2.94 mmHg and the mean central corneal thickness was 530.40 ± 36.87 microns.

**Figure 1:**
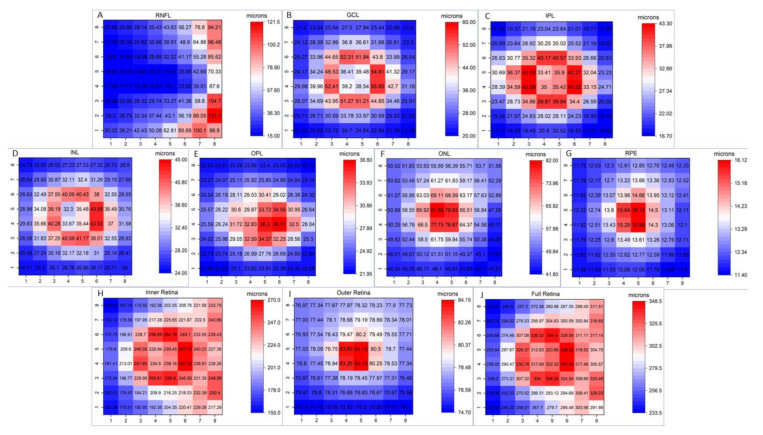
Color thickness map of the different retinal layers in this study (n = 300). The numbers inside the cells indicate mean thickness in microns. Abbreviations: (**A**) RNFL: retinal nerve fiber layer; (**B**) GCL: ganglion cell layer; (**C**) IPL: inner plexiform layer; (**D**) INL: inner nuclear layer; (**E**) OPL: outer plexiform layer; (**F**) ONL: outer nuclear layer; (**G**) RPE: retinal pigmentary epithelium; (**H**) INNER: inner retina; (**I**) OUTER: outer retina; (**J**) RETINA: full retina.

Figure 1 shows the mean thickness values of the different macular layers in all the cells for the 300 eyes.

Supplementary Tables S1–S10 provide the 1st, 5th and 95th percentile thickness values for each macular layer in the 64 cells contained in PPA. While the RNFL was thicker in the cells near the optic disk, GCL, IPL and INL were thicker in paracentral cells. OPL thickness was thicker in the paracentral infero-nasal region. The ONL, RPE and the OUTER retinas were thicker in central cells. The INNER and full RETINA were thicker in paracentral cells, but also in the cells near the optic disk. Layer thickness progressively decreased with eccentricity, except for the RNFL thickness, which was thicker in the peripheral than in the central regions (Figure 1).

**Figure 2:**
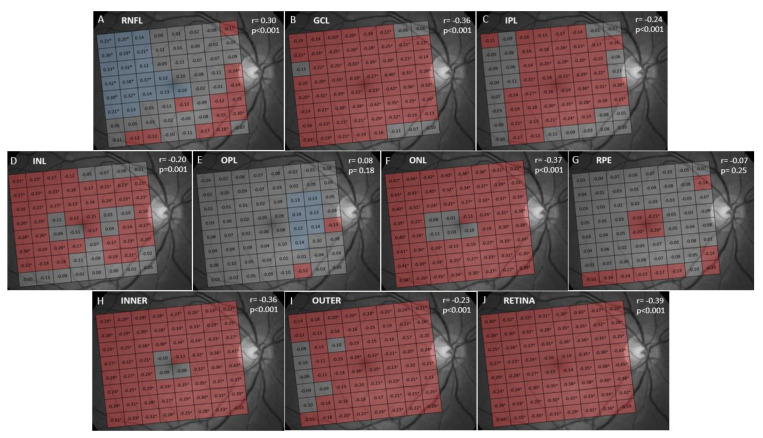
Analysis of correlation between age and retinal layer thickness. The retinal layer analyzed is indicated in the upper left corner of each figure. The Pearson correlation coefficient (r) for the average thickness of the 64 cells of the PPA (and its statistical significance) is indicated in the upper right corner of the figures, and for individual cells within each cell. Cells in blue showed statistically significant positive correlation. Cells in red showed significant negative correlation. Cells in gray did not show a significant correlation. * Cells with *p* < 0.001 for r. Abbreviations: (**A**) RNFL: retinal nerve fiber layer; (**B**) GCL: ganglion cell layer; (**C**) IPL: inner plexiform layer; (**D**) INL: inner nuclear layer; (**E**) OPL: outer plexiform layer; (**F**) ONL: outer nuclear layer; (**G**) RPE: retinal pigmentary epithelium; (**H**) INNER: inner retina; (**I**) OUTER: outer retina; (**J**) RETINA: full retina.

**Figure 3:**
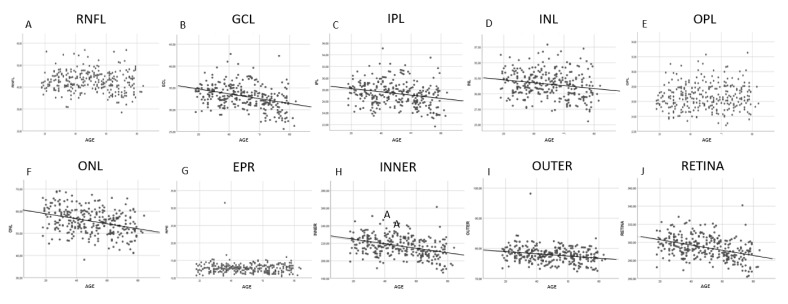
Scatter plot diagrams of the correlation between age and macular layer thickness. Each point represents the average thickness of the 64 cells of PPA for each participant. Line of best fit is only shown in layers with statistically significant correlations. Abbreviations: (**A**) RNFL: retinal nerve fiber layer; (**B**) GCL: ganglion cell layer; (**C**) IPL: inner plexiform layer; (**D**) INL: inner nuclear layer; (**E**) OPL: outer plexiform layer; (**F**) ONL: outer nuclear layer; (**G**) RPE: retinal pigmentary epithelium; (**H**) INNER: inner retina; (**I**) OUTER: outer retina; (**J**) RETINA: full retina.

The results of the correlation analysis between thickness and age are shown in Figure 2 and Figure 3. A significant negative correlation was found between age and thickness in the GCL, IPL, INL, ONL, INNER, OUTER and full RETINA (Figure 3). In contrast, RNFL, OPL and RPE thicknesses did not correlate with age (Figure 3). However, when analyzed by cells, we found that RNFL thickness significantly and negatively correlated with age in the cells near the optic disk and significantly and positively correlated in supero-temporal cells. OPL thickness correlated significantly and positively with age in pericentral nasal cells and RPE thickness showed a significant negative correlation with age in central cells (Figure 2). The negative correlation found in the GCL, IPL, INL, ONL, INNER, OUTER and full RETINA was observed in most cells. However, age had the strongest negative impact on ONL thickness (r = −0.37; *p* < 0.001).

**Figure 4:**
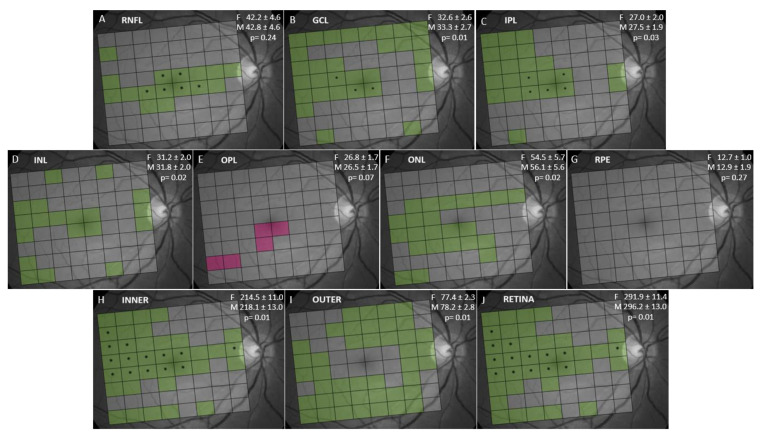
Analysis of retinal layer thickness between sexes using the Student’s test for independent samples. The retinal layer analyzed is indicated in the upper left corner of each figure. Mean ± SD of the thickness of all cells of the PPA for the female (F) and male population (M) (and the statistical significance of the comparison between sexes) is indicated in the upper right corner of the figures. Cells in green were significantly thicker in men than in women. Cells in pink were significantly thicker in women than in men. Cells in gray showed no significant differences sexes. * Cells with *p* < 0.001 for difference of means. Abbreviations: (**A**) RNFL: retinal nerve fiber layer; (**B**) GCL: ganglion cell layer; (**C**) IPL: inner plexiform layer; (**D**) INL: inner nuclear layer; (**E**) OPL: outer plexiform layer; (**F**) ONL: outer nuclear layer; (**G**) RPE: retinal pigmentary epithelium; (**H**) INNER: inner retina; (**I**) OUTER: outer retina; (**J**) RETINA: full retina.

When comparing layer thickness between genders (Figure 4, data of each cell not shown) we observed that the thicknesses of the GCL, IPL, INL, ONL, INNER, OUTER and full RETINA were significantly thicker in men than in women. RNFL, OPL and RPE had similar thicknesses in both genders, although RNFL was thicker in men than in women in central cells.

**Figure 5:**
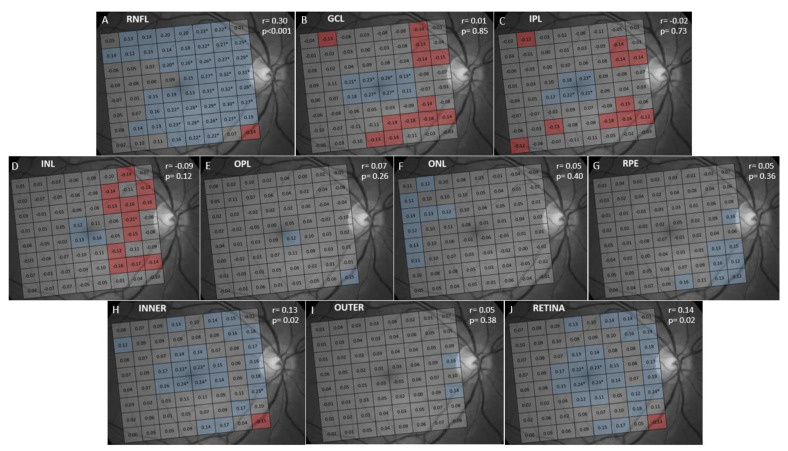
Analysis of correlation between axial length and retinal layer thickness. The retinal layer analyzed is indicated in the upper left corner of each figure. The Pearson correlation coefficient (r) for the average thickness of the 64 cells of the PPA (and its statistical significance) is indicated in the upper right corner of the figures, and for individual cells within each cell. Cells in blue showed statistically significant positive correlation. Cells in red showed significant negative correlation. Cells in gray did not show a significant correlation. * Cells with *p* < 0.001 for r. Abbreviations: (**A**) RNFL: retinal nerve fiber layer; (**B**) GCL: ganglion cell layer; (**C**) IPL: inner plexiform layer; (**D**) INL: inner nuclear layer; (**E**) OPL: outer plexiform layer; (**F**) ONL: outer nuclear layer; (**G**) RPE: retinal pigmentary epithelium; (**H**) INNER: inner retina; (**I**) OUTER: outer retina; (**J**) RETINA: full retina.

**Figure 6:**
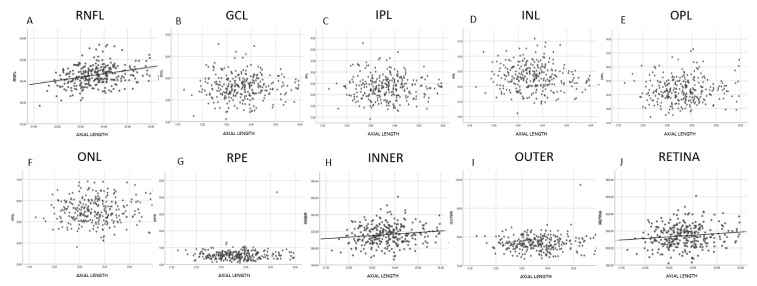
Scatter plot diagrams of the correlation between axial length and macular layer thickness. Each point represents the average thickness of the 64 cells of PPA for each participant. Line of best fit is only shown in layers with statistically significant correlations. Abbreviations: (**A**) RNFL: retinal nerve fiber layer; (**B**) GCL: ganglion cell layer; (**C**) IPL: inner plexiform layer; (**D**) INL: inner nuclear layer; (**E**) OPL: outer plexiform layer; (**F**) ONL: outer nuclear layer; (**G**) RPE: retinal pigmentary epithelium; (**H**) INNER: inner retina; (**I**) OUTER: outer retina; (**J**) RETINA: full retina.

The results of the correlation analysis performed between AXL and retinal layer thickness are shown in Figure 5 and Figure 6. Only RNFL thickness significantly and positively correlated with AXL, while the thicknesses of the other macular layers did not correlate with AXL. We also observed that GCL, IPL and INL thicknesses showed a significantly positive correlation with AXL in central cells and a significant negative correlation in the cells near the optic disk. The INNER and full RETINA thicknesses significantly and positively correlated with AXL, while the OUTER retinal thickness was not significantly correlated with AXL.

## 4. Discussion

SD-OCT devices calculate retinal layer thickness parameters in microns. It is useful to compare the thickness results of a single patient with those of the healthy population which the patient ethnically belongs to.

Comparisons are usually displayed automatically according to a red–yellow–green–white color-code representation to provide the ophthalmologist an idea as to whether the obtained thickness value falls within (green) or beyond (red, yellow or white) the normal range. In the present study, we created a normative database for the 8 × 8 PPA of Spectralis SD-OCT because this protocol lacks a normative database and, thus, does not allow results to be compared. This is the first step to try to define specific patterns of thickness change in macular segmentations on 8 × 8 PPA in different ocular and non-ocular diseases such as glaucoma [4,7], and at different stages of the considered disorder.

We observed that the full RETINA thickness was thicker in the paracentral region and showed a central depression. From the paracentral to the periphery, thickness asymmetrically decreased, which resulted in a thicker thickness in the nasal than in the temporal peripheral region (Figure 1). This thickness distribution is consistent with all previous studies [20,21,22,26,27,28]. Our quantitative thickness data are similar to those reported by other studies carried out in the Caucasian population [20,21], but are higher than the thickness values observed in studies including Asian and Black populations [22,26,27,28], as previously documented [29]. We found only one study, carried out in the Asian population, reporting retinal thickness values in each cell of 8 × 8 PPA [30]. These authors reported similar data to those herein observed, but ethnic populations were different. This could be due to Asian subjects being younger (range 22–41 years) than our Caucasian population (range 18–84 years). Thus, the Asian thickness values did not reflect aging effects. We also documented that RNFL was thicker near the optic disk, GCL, IPL and INL were thicker in the paracentral macula and ONL and RPE were thicker in the central macula. These findings are consistent with previous studies [17,20,21,22].

When we correlated layer thickness with age, we found a significant negative correlation in GCL, IPL, INL, ONL, INNER, OUTER and full RETINA (Figure 3). The layers on which age had the strongest negative impact were ONL and GCL. This ONL thinning with age has been previously documented [21,31]. The age-related decline in GCL thickness has been widely reported in both histological [32,33] and clinical [34,35,36,37] studies. The thickness of inner retinal layers correlated negatively with age, and this finding is also consistent with previous studies [17,21,31,37] that documented GCL, IPL and INL thinning with age. We were unable to document a significant correlation between age and RNFL, OPL and RPE thicknesses as reported by Jorge et al. [37]. However, we found that RNFL underwent significantly decreased thickness with age in several inferonasal and peripapillary cells. This agrees with previous studies [36,38] that report decreased RNFL thickness with age in the most peripheral and nasal macular regions. Conversely, Ooto et al. [34] objectified a significant decreased macular RNFL thickness with age. We also found a significant positive correlation between age and RNFL thickness in the supero-temporal macula. This has been reported previously by other authors [18], who postulated that it could be due to inner limiting membrane thickening with age, which could result in increased measured RNFL thickness [39]. We observed a significant positive correlation between OPL thickness and age in pericentral nasal cells. A previous study [31] also found a significant positive correlation of OPL thickness and age, and a significant negative correlation of ONL thickness and age. These opposite variations in OPL thickness (that thickens) and ONL thickness (that thins) with age could explain why other studies [34,40] found no variations in these layers with age when analyzing the OPL + ONL complex. We do not know if the reason for OPL thickening could be the thickening of Henle’s fiber layer with age [41]. We did not find any significant correlation between RPE thickness and age as previous research works report [31,36,37]. However, we observed a highly significant negative correlation between age and RPE thickness in some central cells which, to our knowledge, has not been reported before. However, we believe that this may be due to loss of RPE cells [32,42]. On the contrary, other authors [35,43] have documented a significant positive correlation between RPE thickness and age, probably due to an increase in residual bodies density, lipofuscin accumulation, drusen development and Bruch’s membrane thickening that occurs with age [44]. Thus, our study did not find this because of the strict inclusion criteria which excluded any eyes with abnormal signs of aging, such as drusen or RPE alterations.

We documented that full RETINA thickness was significantly thicker in men than in women, which is consistent with many previous research works [20,21,28,34,39,45]. Previous studies [20,21,31,46] have reported that GCL, IPL, INL and ONL thicknesses were significantly thicker in men than in women, which falls in line with our results. We found that OPL thickness was similar in both genders, and this finding coincides with other studies [20,21,31] carried out in Caucasian populations. We found that RNFL thickness was similar between genders, which has been previously documented [20,21]. Our study and other previous ones [20,21,31,36] found no gender differences in RPE thickness. We observed a significant positive correlation between AXL and full RETINA thickness, which occurred mainly in central cells. Our results are thus contrary to those of Invernizzi et al. [20], who reported a significantly decreased thickness with AXL in the outer ring of the ETDRS map. Several studies [45,46,47,48] have documented a significant positive correlation of retinal thickness with AXL in the foveal subfield of the ETDRS map and a significant negative correlation in the inner and outer rings. In our study, we failed to document a negative correlation of full RETINA thickness and AXL, which could be due to the different inclusion criteria, as we excluded high myopic eyes (sphere ≥ 5D), while other authors [45,46,47,48] did not establish any limits for refractive defects. We observed that GCL, IPL and INL thicknesses showed a significantly positive correlation with AXL in central cells and a significantly negative correlation in the cells near the optic disk, as similarly reported [47]. It has been postulated that these changes could be due to the mechanical stretching of the retinal tissue that occurs with increasing AXL, that could cause a thinning of the retinal thickness. On the other hand, the flattening of the inner limiting membrane and the centripetal force of the posterior vitreous could result in an elevation of the fovea [49]. Other studies [15,50,51] have documented a significantly reduced thickness of the GCL + IPL complex with AXL. We also found a significantly positive correlation between AXL and RNFL thickness in accordance with previous studies [46,49]. This RNFL thickening could be explained by the traction of the inner limiting membrane that occurs in myopic eyes [51] or by the ocular magnification effects [52]. Finally, we observed that ONL and RPE thicknesses did not correlate with AXL, as described by Invernizzi et al. [20]. However, other authors [45] have documented a significantly negative correlation between AXL and ONL and RPE thicknesses in the extrafoveal rings of the ETDRS map, probably because these authors did not exclude high myopic eyes.

One may argue that a correction for multiple comparisons should be performed in this study. This is a controversial subject because of the risk that false-negative results could increase [53], so we preferred not to apply such adjustments.

### Limitations

This study has some limitations. As it is based on cross-sectional data, the results about thickness changes with age should be interpreted with caution. We included only Caucasian patients, so we cannot generalize our thickness values to other ethnic populations. We excluded high myopic and hyperopic eyes, so this database should not be used for high myopic or hyperopic patients.

## 5. Conclusions

We provide the first normative database of the thicknesses of all macular layers for the 8 × 8 PPA of the SD-OCT Spectralis (Heidelberg Engineering). We also document an effect of age, gender and axial length on the thickness of some different layers as measured by 8 × 8 PPA.

## 6. Future Research

This normative database is the first step to try to define specific patterns of thickness change in macular segmentations on 8 × 8 PPA in different ocular and non-ocular diseases and at different stages of the considered disorder.

## Supplementary Materials

The following are available online at https://www.mdpi.com/2077-0383/9/10/3317/s1, Table S1: 1st, 5th and 95th percentile values of the retinal nerve fiber layer (RNFL) thickness in each of the 64 cells of the 8 × 8 macular grid in the different age groups, Table S2: 1st, 5th and 95th percentiles values of the ganglion cell layer (GCL) thickness in each of the 64 cells of the 8 × 8 macular grid in the different age groups, Table S3: 1st, 5th and 95th percentiles values of the inner plexiform layer (IPL) thickness in each of the 64 cells of the 8 × 8 macular grid in the different age groups, Table S4: 1st, 5th and 95th percentiles values of the inner nuclear layer (INL) thickness in each of the 64 cells of the 8 × 8 macular grid in the different age groups, Table S5: 1st, 5th and 95th percentiles values of the outer plexiform layer (OPL) thickness in each of the 64 cells of the 8 × 8 macular grid in the different age groups, Table S6: 1st, 5th and 95th percentiles values of the outer nuclear layer (ONL) thickness in each of the 64 cells of the 8 × 8 macular grid in the different age groups, Table S7: 1st, 5th and 95th percentiles values of the retinal pigment epithelium (RPE) thickness in each of the 64 cells of the 8 × 8 macular grid in the different age groups, Table S8: 1st, 5th and 95th percentiles values of the INNER retina thickness in each of the 64 cells of the 8 × 8 macular grid in the different age groups, Table S9: 1st, 5th and 95th percentiles values of the OUTER retina thickness in each of the 64 cells of the 8 × 8 macular grid in the different age groups, Table S10: 1st, 5th and 95th percentiles values of the full RETINA thickness in each of the 64 cells of the 8 × 8 macular grid in the different age groups.
