# Supplementary material for: Normative Database for All Retinal Layer Thicknesses Using SD-OCT Posterior Pole Algorithm and the Effects of Age, Gender and Axial Lenght
Source: J Clin Med. 2020 Oct 15;9(10):3317. doi: 10.3390/jcm9103317 (PMC7602827; doi:10.3390/jcm9103317)
Supplement: Supplementary file 1 [file jcm-09-03317-s001.pdf]

Supplementary

Table S1. 1st, 5th and 95th percentile values of the retinal nerve fiber layer (RNFL) thickness in each of the 64 cells of the 8 × 8 macular grid in the different age groups.

RNFL	1.1			1.2			1.3			1.4		
	**p1**	**p5**	**p95**	**p1**	**p5**	**p95**	**p1**	**p5**	**p95**	**p1**	**p5**	**p95**
18–29	21.00	22.55	42.90	28.00	28.00	52.00	31.00	32.55	60.35	35.00	36.00	72.80
30–39	20.00	21.55	37.45	23.00	25.10	46.90	29.00	31.10	56.00	32.00	36.00	71.45
40–49	20.00	21.55	41.00	24.00	24.00	53.90	28.00	30.00	64.45	30.00	35.65	71.90
50–59	18.00	21.00	39.80	24.00	25.00	48.00	31.00	31.00	59.00	38.00	39.00	68.25
60–69	18.00	19.55	38.45	20.00	23.10	50.00	17.00	27.00	62.45	23.00	31.65	74.60
70–85	18.00	19.55	45.70	25.00	25.00	55.45	26.00	28.55	58.00	31.00	33.10	72.35
	1.5			1.6			1.7			1.8		
	p1	p5	p95	p1	p5	p95	p1	p5	p95	p1	p5	p95
18–29	40.00	43.55	95.25	54.00	60.10	114.90	77.00	83.20	129.35	51.00	56.65	117.45
30–39	35.00	41.10	89.70	39.00	52.00	113.25	63.00	71.10	129.70	46.00	56.20	112.45
40–49	39.00	44.65	87.90	54.00	56.65	116.80	73.00	77.85	127.90	59.00	67.55	130.75
50–59	43.00	46.10	93.60	58.00	59.10	114.45	67.00	73.65	126.35	39.00	53.55	121.90
60–69	36.00	39.55	96.90	47.00	47.55	116.45	52.00	63.85	114.70	38.00	47.75	109.00
70–85	35.00	37.55	93.45	41.00	45.55	116.70	43.00	56.30	127.80	53.00	57.10	130.75
	2.1			2.2			2.3			2.4		
	p1	p5	p95	p1	p5	p95	p1	p5	p95	p1	p5	p95
18–29	18.00	18.55	36.00	19.00	20.65	39.45	26.00	26.55	43.45	31.00	31.00	47.45
30–39	18.00	18.00	32.00	20.00	21.55	38.00	26.00	27.00	45.90	26.00	29.55	48.90
40–49	19.00	19.00	37.35	20.00	22.00	38.90	23.00	26.10	43.00	30.00	30.55	47.00
50–59	13.00	18.55	32.00	16.00	22.55	34.00	26.00	26.55	41.80	29.00	30.55	45.80
60–69	13.00	18.00	37.45	12.00	19.55	38.80	13.00	22.00	42.00	16.00	26.65	47.45
70–85	19.00	20.10	37.35	20.00	21.10	39.90	23.00	25.10	46.35	24.00	28.55	51.35
	2.5			2.6			2.7			2.8		
	p1	p5	p95	p1	p5	p95	p1	p5	p95	p1	p5	p95
18–29	34.00	34.00	55.25	41.00	44.00	82.40	59.00	64.10	130.20	84.00	100.65	156.00
30–39	29.00	31.65	59.00	33.00	40.50	83.60	44.00	51.40	122.45	70.00	87.95	145.35
40–49	34.00	35.00	53.45	40.00	41.00	75.90	49.00	58.65	124.35	68.00	101.75	156.00
50–59	34.00	37.10	53.45	41.00	43.55	79.00	59.00	62.65	122.80	75.00	93.20	152.05
60–69	30.00	31.00	53.90	38.00	38.55	78.00	51.00	54.10	121.90	74.00	82.10	140.45
70–85	27.00	32.55	54.45	34.00	38.30	75.45	45.00	47.55	113.95	64.00	75.40	148.70
	3.1			3.2			3.3			3.4		
	p1	p5	p95	p1	p5	p95	p1	p5	p95	p1	p5	p95
18–29	15.00	15.55	25.45	16.00	18.00	27.00	21.00	21.55	32.00	23.00	23.55	34.45
30–39	16.00	16.00	27.00	17.00	18.00	30.45	21.00	21.55	32.00	24.00	24.00	37.45
40–49	16.00	16.00	27.45	16.00	18.10	30.45	21.00	22.00	35.45	24.00	24.55	37.00
50–59	15.00	16.55	24.90	18.00	18.00	27.90	20.00	22.00	33.00	24.00	25.55	36.45
60–69	15.00	16.55	30.35	17.00	18.55	31.35	18.00	20.55	33.25	23.00	24.00	35.90
70–85	17.00	17.55	29.90	17.00	18.00	32.00	16.00	18.65	33.00	17.00	19.75	35.90
	3.5			3.6			3.7			3.8		
	p1	p5	p95	p1	p5	p95	p1	p5	p95	p1	p5	p95
18–29	26.00	27.10	38.90	31.00	32.65	51.80	45.00	45.55	78.25	75.00	80.65	149.50
30–39	23.00	26.65	42.00	29.00	31.55	53.90	37.00	40.20	81.45	51.00	63.55	146.15
40–49	25.00	28.00	41.45	33.00	34.00	51.90	40.00	46.55	76.15	55.00	75.55	146.40
50–59	26.00	28.10	40.45	33.00	34.55	50.00	45.00	49.10	76.70	74.00	78.30	137.60
60–69	26.00	26.00	41.45	32.00	32.00	51.45	41.00	41.55	81.45	60.00	62.55	137.90
70–85	16.00	20.40	40.45	20.00	25.40	52.45	33.00	35.65	76.70	47.00	52.75	136.35
	4.1			4.2			4.3			4.4		
	p1	p5	p95	p1	p5	p95	p1	p5	p95	p1	p5	p95
18–29	14.00	14.00	20.45	14.00	15.00	19.00	14.00	15.00	20.45	13.00	13.55	17.90
30–39	14.00	14.00	20.45	14.00	15.00	20.00	15.00	15.55	22.00	13.00	14.00	19.00
40–49	14.00	14.55	22.45	15.00	15.00	22.45	14.00	15.00	23.45	13.00	13.55	19.00
50–59	14.00	14.00	21.00	15.00	15.00	22.00	16.00	16.00	22.00	14.00	14.55	19.00
60–69	14.00	15.00	22.00	15.00	15.00	22.00	15.00	16.00	23.00	14.00	14.00	20.00

70–85	14.00	15.55	23.45	15.00	15.00	23.00	13.00	16.00	23.00	13.00	14.00	19.00
		4.5			4.6			4.7			4.8	
	p1	p5	p95	p1	p5	p95	p1	p5	p95	p1	p5	p95
18–29	13.00	13.00	18.00	17.00	18.00	28.45	29.00	31.55	49.45	46.00	52.65	88.90
30–39	13.00	14.00	19.00	16.00	18.55	30.35	29.00	32.00	50.90	45.00	48.65	91.90
40–49	12.00	13.55	19.45	15.00	19.55	29.35	28.00	31.00	50.70	43.00	51.00	92.70
50–59	13.00	14.00	19.45	19.00	20.55	32.25	30.00	33.00	47.45	55.00	56.10	94.15
60–69	13.00	14.00	19.00	18.00	18.55	29.00	30.00	30.55	47.45	47.00	47.00	89.60
70–85	13.00	13.00	19.45	17.00	17.00	28.90	24.00	27.75	50.00	36.00	41.55	86.90
**RNFL**		5.1			5.2			5.3			5.4	
	p1	p5	p95	p1	p5	p95	p1	p5	p95	p1	p5	p95
18–29	13.00	14.00	21.45	13.00	13.55	21.00	13.00	13.00	16.00	12.00	13.00	17.45
30–39	12.00	13.10	21.00	13.00	13.55	21.90	13.00	13.00	17.00	12.00	13.00	18.00
40–49	12.00	13.55	22.00	13.00	13.55	21.90	12.00	13.00	18.45	13.00	13.55	19.45
50–59	15.00	15.00	24.45	13.00	13.00	21.45	13.00	13.00	19.00	13.00	13.00	18.00
60–69	15.00	16.00	24.00	13.00	15.55	23.00	14.00	14.00	18.45	13.00	14.00	17.45
70–85	13.00	14.00	24.00	14.00	16.55	24.00	13.00	13.55	21.45	13.00	13.00	18.00
		5.5			5.6			5.7			5.8	
	p1	p5	p95	p1	p5	p95	p1	p5	p95	p1	p5	p95
18–29	14.00	14.00	18.45	19.00	21.55	33.45	33.00	35.00	53.90	48.00	57.75	90.35
30–39	12.00	13.55	19.45	19.00	20.00	32.00	32.00	32.55	52.45	43.00	52.65	96.45
40–49	14.00	14.00	21.45	19.00	20.00	32.45	31.00	34.55	58.45	48.00	58.20	103.00
50–59	13.00	14.00	19.00	20.00	21.00	32.00	31.00	35.55	51.45	55.00	58.55	89.45
60–69	13.00	13.55	19.45	19.00	20.00	32.00	30.00	31.55	52.90	47.00	49.00	87.95
70–85	14.00	14.00	22.00	18.00	19.55	35.00	27.00	30.10	58.45	41.00	46.75	96.15
		6.1			6.2			6.3			6.4	
	p1	p5	p95	p1	p5	p95	p1	p5	p95	p1	p5	p95
18–29	14.00	14.00	18.00	14.00	14.00	19.00	14.00	15.00	22.45	18.00	20.10	30.45
30–39	14.00	14.00	19.45	14.00	14.00	19.00	15.00	16.00	25.45	19.00	20.00	31.45
40–49	13.00	14.00	20.00	15.00	15.00	24.45	16.00	16.00	28.90	20.00	21.00	35.15
50–59	14.00	14.00	20.00	14.00	14.55	24.00	16.00	17.00	24.45	21.00	22.55	34.00
60–69	14.00	14.00	21.00	15.00	15.55	22.45	16.00	17.00	25.00	20.00	20.55	29.45
70–85	15.00	16.00	22.45	15.00	15.00	23.00	17.00	17.00	25.45	17.00	18.10	34.45
		6.5			6.6			6.7			6.8	
	p1	p5	p95	p1	p5	p95	p1	p5	p95	p1	p5	p95
18–29	23.00	25.55	38.00	28.00	34.55	48.45	38.00	45.00	65.00	49.00	60.60	112.35
30–39	25.00	25.55	38.00	29.00	29.00	51.45	34.00	40.20	73.90	52.00	55.75	113.40
40–49	25.00	26.55	43.90	32.00	33.55	54.00	28.00	44.00	75.90	25.00	63.10	123.45
50–59	25.00	26.10	41.35	32.00	32.55	52.45	41.00	46.00	70.45	62.00	66.10	115.90
60–69	24.00	25.10	39.00	31.00	32.55	51.00	41.00	43.55	68.35	58.00	59.55	116.50
70–85	22.00	23.55	43.90	29.00	29.00	54.00	36.00	40.55	74.80	47.00	55.65	115.50
		7.1			7.2			7.3			7.4	
	p1	p5	p95	p1	p5	p95	p1	p5	p95	p1	p5	p95
18–29	13.00	13.00	19.00	14.00	15.00	24.00	17.00	18.55	30.00	23.00	24.00	36.00
30–39	13.00	13.00	24.45	14.00	15.00	28.35	17.00	18.55	31.45	21.00	22.00	38.35
40–49	11.00	14.00	23.00	16.00	16.00	27.90	18.00	19.00	35.45	25.00	25.55	45.80
50–59	13.00	14.00	24.35	14.00	15.55	26.35	17.00	19.55	34.35	22.00	24.55	41.00
60–69	13.00	14.55	25.45	15.00	16.00	28.00	18.00	19.55	33.00	22.00	22.55	38.35
70–85	14.00	15.00	28.00	15.00	16.55	29.45	18.00	20.55	34.00	20.00	22.65	41.00
		7.5			7.6			7.7			7.8	
	p1	p5	p95	p1	p5	p95	p1	p5	p95	p1	p5	p95
18–29	30.00	31.55	45.45	37.00	39.55	56.00	47.00	49.20	80.90	64.00	70.55	130.45
30–39	27.00	28.55	47.45	33.00	36.00	59.90	43.00	45.00	84.15	56.00	60.00	128.90
40–49	30.00	32.55	56.60	38.00	40.00	67.25	47.00	51.20	96.95	71.00	73.55	134.70
50–59	28.00	29.55	49.45	36.00	36.55	64.80	46.00	47.00	96.75	71.00	72.65	143.80
60–69	29.00	29.55	46.45	34.00	37.00	61.80	43.00	46.10	85.45	58.00	66.20	124.80
70–85	25.00	28.65	52.00	33.00	34.55	65.15	40.00	42.55	83.45	51.00	59.40	126.20
		8.1			8.2			8.3			8.4	
	p1	p5	p95	p1	p5	p95	p1	p5	p95	p1	p5	p95
18–29	14.00	15.00	26.45	16.00	17.55	30.00	20.00	22.00	35.45	24.00	28.55	42.45
30–39	14.00	14.55	25.00	16.00	17.00	31.90	18.00	19.00	40.45	22.00	24.10	47.90
40–49	13.00	15.00	25.45	11.00	15.20	32.00	12.00	19.00	41.45	20.00	27.00	54.70
50–59	11.00	14.65	26.45	15.00	18.10	32.00	21.00	21.00	38.00	25.00	27.00	46.00

60–69	15.00	16.00	27.45	17.00	18.55	32.45	21.00	22.00	37.45	25.00	26.00	46.00
70–85	16.00	16.55	30.90	17.00	18.00	35.45	20.00	22.00	40.45	24.00	26.10	48.00
		8.5			8.6			8.7			8.8	
	p1	p5	p95	p1	p5	p95	p1	p5	p95	p1	p5	p95
18–29	30.00	36.00	55.45	39.00	45.10	78.70	52.00	61.55	105.90	67.00	73.75	123.45
30–39	26.00	27.65	56.90	32.00	36.55	75.45	40.00	49.10	101.90	63.00	68.85	119.15
40–49	26.00	33.00	70.90	35.00	42.55	88.80	47.00	54.20	109.90	59.00	63.55	127.45
50–59	24.00	32.00	64.90	26.00	40.10	84.60	38.00	52.00	112.45	66.00	69.30	124.90
60–69	30.00	31.55	57.15	34.00	40.10	73.00	41.00	49.00	98.45	44.00	58.75	126.70
70–85	26.00	29.10	55.45	32.00	36.55	72.00	37.00	45.10	102.90	49.00	60.00	117.70

Table S2. 1st, 5th and 95th percentiles values of the ganglion cell layer (GCL) thickness in each of the 64 cells of the 8 × 8 macular grid in the different age groups.

GCL		**1.1**			**1.2**			**1.3**			**1.4**	
	**p1**	**p5**	**p95**	**p1**	**p5**	**p95**	**p1**	**p5**	**p95**	**p1**	**p5**	**p95**
18–29	18.00	19.00	25.45	20.00	20.00	26.45	21.00	21.00	27.00	21.00	22.00	28.00
30–39	17.00	18.00	24.00	19.00	19.00	26.00	20.00	20.00	27.45	21.00	22.00	29.90
40–49	18.00	19.00	27.90	20.00	20.55	27.90	21.00	21.55	28.45	22.00	22.00	29.45
50–59	13.00	18.00	25.00	16.00	19.00	27.00	20.00	20.55	28.00	20.00	20.55	29.00
60–69	12.00	15.55	25.45	15.00	16.55	25.00	12.00	18.20	27.00	14.00	18.55	27.00
70–85	14.00	15.55	25.00	17.00	17.00	27.45	18.00	19.00	29.00	19.00	20.00	31.00
		1.5			1.6			1.7			1.8	
	p1	p5	p95	p1	p5	p95	p1	p5	p95	p1	p5	p95
18–29	21.00	21.55	29.45	17.00	18.00	27.45	17.00	17.55	26.00	17.00	19.00	24.45
30–39	20.00	20.55	27.45	19.00	19.00	27.45	15.00	17.00	26.00	18.00	18.55	24.90
40–49	21.00	22.00	30.45	18.00	19.55	29.35	17.00	17.55	27.45	17.00	18.00	25.45
50–59	20.00	20.55	29.35	18.00	18.55	28.00	14.00	16.55	26.45	17.00	18.00	25.00
60–69	19.00	20.55	29.00	20.00	20.00	28.35	16.00	17.55	26.90	12.00	17.00	26.45
70–85	19.00	19.55	30.00	16.00	17.65	27.45	15.00	16.55	25.45	17.00	17.55	26.00
		2.1			2.2			2.3			2.4	
	p1	p5	p95	p1	p5	p95	p1	p5	p95	p1	p5	p95
18–29	20.00	21.00	28.00	22.00	24.00	30.45	26.00	26.55	36.45	25.00	28.55	40.45
30–39	19.00	19.55	27.00	21.00	23.00	31.00	23.00	26.55	35.90	27.00	29.10	40.00
40–49	20.00	22.00	29.00	23.00	25.00	33.00	28.00	28.00	38.00	30.00	30.00	40.90
50–59	13.00	18.55	30.00	14.00	22.10	32.45	22.00	26.00	36.45	29.00	29.00	39.45
60–69	11.00	18.55	26.90	10.00	19.75	29.45	9.00	21.50	34.45	14.00	25.75	37.45
70–85	18.00	19.00	29.35	21.00	21.00	32.35	24.00	25.00	38.00	26.00	27.00	44.25
		2.5			2.6			2.7			2.8	
	p1	p5	p95	p1	p5	p95	p1	p5	p95	p1	p5	p95
18–29	29.00	30.00	42.45	25.00	26.55	36.45	21.00	21.55	30.45	17.00	17.00	26.45
30–39	26.00	29.10	40.45	21.00	25.10	36.00	19.00	20.00	30.45	16.00	17.55	25.00
40–49	30.00	31.55	40.90	27.00	27.55	38.90	20.00	21.55	32.35	17.00	18.00	28.00
50–59	28.00	29.55	40.45	24.00	24.55	36.00	20.00	21.00	32.00	13.00	15.20	28.45
60–69	22.00	25.65	37.45	22.00	23.55	35.00	18.00	18.55	29.45	17.00	17.00	24.45
70–85	27.00	27.00	42.35	23.00	24.00	38.00	17.00	18.55	33.80	16.00	16.55	28.45
		3.1			3.2			3.3			3.4	
	p1	p5	p95	p1	p5	p95	p1	p5	p95	p1	p5	p95
18–29	21.00	24.55	32.00	28.00	30.00	40.00	37.00	38.00	51.00	43.00	44.65	60.00
30–39	21.00	24.10	33.00	29.00	30.55	41.45	39.00	39.00	50.45	43.00	46.00	59.00
40–49	24.00	25.55	34.00	29.00	31.55	41.00	40.00	40.55	52.00	45.00	48.00	60.45
50–59	22.00	23.10	34.45	27.00	30.10	41.45	38.00	38.55	51.45	41.00	46.55	59.90
60–69	22.00	22.55	31.45	26.00	27.55	38.90	30.00	35.00	48.90	40.00	41.55	56.90
70–85	21.00	22.00	35.90	25.00	26.55	42.45	33.00	33.00	50.00	31.00	37.55	57.00
		3.5			3.6			3.7			3.8	
	p1	p5	p95	p1	p5	p95	p1	p5	p95	p1	p5	p95
18–29	44.00	45.00	60.00	35.00	40.00	51.45	27.00	30.00	40.00	21.00	22.00	32.00
30–39	41.00	44.00	60.80	36.00	38.00	55.00	27.00	28.55	42.00	21.00	21.55	33.45
40–49	43.00	48.10	60.45	41.00	42.00	54.80	29.00	30.65	45.15	22.00	22.00	35.90
50–59	41.00	44.55	60.00	33.00	38.10	54.90	26.00	30.55	40.45	19.00	19.00	32.45
60–69	39.00	42.00	56.45	34.00	35.00	49.45	25.00	27.10	39.00	19.00	19.55	29.45
70–85	36.00	37.55	55.00	31.00	32.55	51.90	25.00	26.55	42.00	20.00	20.00	32.45
		4.1			4.2			4.3			4.4	

	p1	p5	p95	p1	p5	p95	p1	p5	p95	p1	p5	p95
18–29	24.00	24.55	36.00	32.00	33.00	49.00	47.00	48.00	61.45	26.00	32.00	49.35
30–39	26.00	26.00	37.00	34.00	35.55	48.35	45.00	47.10	61.35	31.00	31.55	51.90
40–49	22.00	24.55	38.00	31.00	34.10	50.35	41.00	47.00	62.35	19.00	25.30	51.35
50–59	21.00	24.10	36.45	32.00	34.00	49.00	48.00	48.00	61.45	27.00	31.10	48.45
60–69	18.00	22.00	33.00	24.00	28.20	45.45	40.00	42.55	56.90	22.00	28.00	47.45
70–85	19.00	20.55	39.70	22.00	26.55	48.35	35.00	38.00	59.00	23.00	25.55	47.45
		4.5			4.6			4.7			4.8	
	p1	p5	p95	p1	p5	p95	p1	p5	p95	p1	p5	p95
18–29	30.00	31.00	53.35	50.00	52.00	65.00	37.00	39.00	49.00	26.00	27.55	37.45
30–39	23.00	29.10	49.00	50.00	50.00	66.45	34.00	36.55	52.45	25.00	25.55	41.45
40–49	26.00	26.00	49.90	41.00	48.40	65.70	40.00	41.00	54.15	27.00	28.00	42.45
50–59	25.00	28.65	52.45	50.00	51.00	65.00	35.00	36.55	50.00	24.00	25.00	37.00
60–69	22.00	25.55	46.45	43.00	45.10	60.45	32.00	33.55	46.45	25.00	25.00	36.00
70–85	23.00	23.55	45.90	39.00	43.00	59.45	32.00	32.00	47.35	21.00	23.00	35.45
GCL		5.1			5.2			5.3			5.4	
	p1	p5	p95	p1	p5	p95	p1	p5	p95	p1	p5	p95
18–29	18.00	20.00	32.45	25.00	25.55	46.45	41.00	43.20	58.00	26.00	30.00	46.35
30–39	19.00	19.55	33.90	24.00	27.10	44.00	41.00	41.55	59.00	23.00	27.00	49.00
40–49	20.00	20.00	34.45	26.00	27.55	48.90	36.00	43.10	59.90	24.00	26.00	52.70
50–59	18.00	18.55	30.90	25.00	26.55	47.00	42.00	43.00	58.35	23.00	28.10	47.00
60–69	16.00	16.55	29.45	20.00	21.55	40.45	34.00	37.55	54.00	22.00	24.65	42.90
70–85	16.00	16.55	30.45	20.00	21.00	43.35	30.00	31.10	55.35	15.00	20.10	49.15
		5.5			5.6			5.7			5.8	
	p1	p5	p95	p1	p5	p95	p1	p5	p95	p1	p5	p95
18–29	32.00	33.55	51.90	49.00	50.00	62.45	34.00	36.55	49.00	25.00	25.55	37.00
30–39	24.00	29.00	48.45	46.00	49.55	65.45	32.00	33.55	50.90	22.00	24.55	37.45
40–49	21.00	30.65	53.45	43.00	50.55	64.70	34.00	36.10	50.70	22.00	23.55	38.00
50–59	29.00	30.65	50.90	48.00	50.55	63.45	35.00	36.55	50.00	24.00	24.00	36.45
60–69	27.00	27.55	45.45	43.00	46.10	60.45	32.00	33.55	46.45	23.00	24.00	33.45
70–85	17.00	23.40	51.80	37.00	41.10	57.45	28.00	30.00	46.35	22.00	22.00	33.45
		6.1			6.2			6.3			6.4	
	p1	p5	p95	p1	p5	p95	p1	p5	p95	p1	p5	p95
18–29	18.00	19.55	32.90	23.00	28.00	42.00	37.00	39.10	54.00	44.00	46.10	60.90
30–39	16.00	21.55	32.00	26.00	27.55	42.35	35.00	38.55	54.25	44.00	46.10	61.80
40–49	20.00	20.55	34.35	27.00	28.00	44.80	35.00	37.65	55.25	43.00	45.10	62.35
50–59	20.00	20.00	30.45	28.00	28.00	42.00	40.00	40.55	54.00	48.00	48.00	62.00
60–69	20.00	20.55	30.90	24.00	25.10	37.45	33.00	36.10	49.00	41.00	43.55	58.45
70–85	18.00	18.55	32.45	23.00	24.55	39.45	29.00	32.55	51.00	33.00	39.55	57.45
		6.5			6.6			6.7			6.8	
	p1	p5	p95	p1	p5	p95	p1	p5	p95	p1	p5	p95
18–29	44.00	47.00	60.00	37.00	38.00	51.35	28.00	29.55	38.45	22.00	22.00	31.00
30–39	43.00	45.55	62.00	37.00	38.10	53.90	28.00	28.00	41.90	21.00	21.55	32.90
40–49	41.00	44.75	62.35	36.00	38.55	54.35	26.00	28.55	41.45	20.00	21.65	33.80
50–59	47.00	47.00	61.00	38.00	38.55	52.45	29.00	30.00	41.45	22.00	22.00	33.45
60–69	41.00	43.10	56.45	33.00	36.65	48.90	28.00	28.55	37.00	19.00	22.10	31.00
70–85	30.00	38.10	55.45	29.00	32.55	50.00	25.00	26.00	38.00	19.00	21.55	31.00
		7.1			7.2			7.3			7.4	
	p1	p5	p95	p1	p5	p95	p1	p5	p95	p1	p5	p95
18–29	19.00	21.55	28.00	24.00	25.00	34.00	29.00	29.00	38.45	31.00	32.00	43.35
30–39	19.00	20.00	29.00	24.00	24.00	34.90	26.00	27.00	39.45	30.00	31.10	44.45
40–49	21.00	22.00	32.00	24.00	24.55	36.45	28.00	28.55	43.00	31.00	31.55	46.00
50–59	17.00	19.00	29.45	21.00	23.00	34.35	28.00	29.00	39.00	33.00	33.00	44.45
60–69	19.00	20.00	26.45	23.00	23.55	31.00	28.00	28.00	37.45	29.00	30.00	41.45
70–85	17.00	18.55	29.00	23.00	23.00	34.45	25.00	25.55	37.90	24.00	27.00	42.45
		7.5			7.6			7.7			7.8	
	p1	p5	p95	p1	p5	p95	p1	p5	p95	p1	p5	p95
18–29	29.00	31.55	43.45	26.00	27.55	38.45	22.00	24.00	32.90	20.00	20.00	30.00
30–39	31.00	31.00	45.00	26.00	26.55	38.00	22.00	22.00	31.45	18.00	19.55	27.45
40–49	31.00	32.10	45.45	26.00	28.00	40.00	21.00	22.00	33.45	18.00	19.00	29.00
50–59	31.00	33.10	44.45	25.00	28.00	39.00	21.00	22.55	34.00	19.00	20.00	28.90
60–69	28.00	31.00	40.90	26.00	27.10	35.90	20.00	23.00	30.00	17.00	18.55	26.00
70–85	24.00	25.00	41.90	20.00	24.55	36.45	21.00	21.00	31.00	17.00	18.55	27.45

	8.1			8.2			8.3			8.4		
	p1	p5	p95	p1	p5	p95	p1	p5	p95	p1	p5	p95
18–29	18.00	19.00	25.00	20.00	20.55	28.00	21.00	21.00	29.45	22.00	24.00	32.45
30–39	17.00	17.55	25.00	20.00	20.00	28.00	21.00	21.00	31.00	22.00	23.00	32.45
40–49	15.00	17.55	27.00	15.00	19.55	29.45	12.00	21.55	32.45	15.00	23.00	32.90
50–59	16.00	16.55	25.45	13.00	19.10	27.45	20.00	21.55	30.45	22.00	23.55	33.45
60–69	18.00	18.00	24.00	19.00	20.55	26.45	22.00	22.00	29.00	22.00	22.55	31.35
70–85	17.00	17.00	26.00	19.00	19.00	28.45	20.00	20.00	31.45	22.00	22.00	33.90
	8.5			8.6			8.7			8.8		
	p1	p5	p95	p1	p5	p95	p1	p5	p95	p1	p5	p95
18–29	23.00	24.55	32.90	21.00	22.00	31.00	19.00	19.00	28.90	16.00	17.00	28.00
30–39	22.00	23.55	33.90	21.00	22.00	31.45	18.00	19.00	26.45	18.00	18.55	26.45
40–49	19.00	23.55	33.45	20.00	20.55	30.45	18.00	19.00	27.35	15.00	17.00	27.00
50–59	21.00	24.00	34.80	18.00	20.65	30.00	19.00	19.55	28.45	19.00	19.00	27.00
60–69	22.00	23.00	33.00	20.00	21.00	29.45	19.00	19.55	29.00	18.00	18.00	27.45
70–85	21.00	21.00	33.45	19.00	20.00	30.00	18.00	18.55	26.00	16.00	18.00	27.45

Table S3. 1st, 5th and 95th percentiles values of the inner plexiform layer (IPL) thickness in each of the 64 cells of the 8 × 8 macular grid in the different age groups.

IPL	**1.1**			**1.2**			**1.3**			**1.4**		
	**p1**	**p5**	**p95**	**p1**	**p5**	**p95**	**p1**	**p5**	**p95**	**p1**	**p5**	**p95**
18–29	12.00	14.00	21.00	14.00	15.00	22.45	17.00	17.00	23.90	16.00	16.55	25.00
30–39	13.00	13.00	21.00	12.00	13.00	22.45	14.00	14.55	22.90	15.00	16.55	25.00
40–49	13.00	13.00	22.00	14.00	15.55	24.00	14.00	16.55	26.00	18.00	18.55	26.00
50–59	13.00	13.00	21.00	14.00	14.00	23.45	14.00	14.55	23.00	14.00	17.00	25.00
60–69	13.00	13.00	21.00	13.00	14.00	22.00	13.00	15.55	23.45	16.00	17.00	23.00
70–85	11.00	13.00	22.00	12.00	15.00	21.45	14.00	14.55	24.00	14.00	15.55	27.00
	1.5			1.6			1.7			1.8		
	p1	p5	p95	p1	p5	p95	p1	p5	p95	p1	p5	p95
18–29	15.00	17.00	25.00	12.00	13.10	24.45	11.00	12.00	21.00	13.00	13.00	23.00
30–39	14.00	15.00	24.00	13.00	13.55	22.90	11.00	12.00	22.45	12.00	12.00	20.45
40–49	15.00	17.55	26.45	12.00	14.55	22.45	12.00	12.55	22.45	10.00	11.55	22.90
50–59	14.00	17.55	25.00	12.00	14.55	24.35	11.00	12.00	21.45	12.00	13.00	23.35
60–69	13.00	16.00	26.35	15.00	15.00	25.45	10.00	12.55	23.90	11.00	12.00	25.90
70–85	15.00	16.00	26.00	11.00	13.10	24.90	11.00	12.00	21.45	12.00	12.00	24.45
	2.1			2.2			2.3			2.4		
	p1	p5	p95	p1	p5	p95	p1	p5	p95	p1	p5	p95
18–29	15.00	15.00	23.00	19.00	19.00	27.90	21.00	21.00	30.00	22.00	24.00	35.00
30–39	14.00	15.00	22.90	16.00	17.00	26.90	19.00	20.55	29.00	21.00	22.55	32.45
40–49	16.00	16.00	23.00	18.00	20.00	26.90	20.00	21.55	31.00	23.00	26.00	34.45
50–59	13.00	14.00	25.90	16.00	17.55	27.00	20.00	21.10	30.00	23.00	23.00	32.00
60–69	14.00	14.55	22.45	16.00	17.55	24.45	18.00	20.00	27.45	21.00	21.55	30.00
70–85	13.00	14.55	23.90	16.00	16.55	27.45	19.00	19.55	31.45	21.00	22.00	35.45
	2.5			2.6			2.7			2.8		
	p1	p5	p95	p1	p5	p95	p1	p5	p95	p1	p5	p95
18–29	24.00	24.55	33.00	20.00	21.00	29.00	15.00	15.55	24.00	10.00	12.55	24.00
30–39	22.00	23.00	33.45	17.00	18.55	29.00	13.00	14.00	25.00	9.00	11.55	21.00
40–49	26.00	26.00	33.45	21.00	21.00	28.90	15.00	16.55	26.00	12.00	13.00	23.00
50–59	24.00	24.00	33.00	19.00	20.00	29.45	15.00	15.00	26.00	12.00	12.00	23.00
60–69	20.00	21.55	31.90	18.00	19.00	27.45	12.00	13.55	24.00	12.00	13.00	22.35
70–85	22.00	22.00	33.45	17.00	19.00	28.45	11.00	14.55	27.45	10.00	11.55	25.00
	3.1			3.2			3.3			3.4		
	p1	p5	p95	p1	p5	p95	p1	p5	p95	p1	p5	p95
18–29	16.00	18.10	27.00	24.00	24.55	33.45	30.00	30.55	40.45	34.00	35.55	45.90
30–39	16.00	18.55	30.00	23.00	24.55	34.45	29.00	30.00	38.45	35.00	35.55	44.00
40–49	19.00	19.55	28.45	25.00	26.00	34.00	31.00	31.00	42.00	37.00	37.55	46.45
50–59	16.00	17.65	28.45	23.00	24.55	33.45	29.00	30.00	40.00	33.00	36.55	46.45
60–69	18.00	19.00	28.00	23.00	24.00	32.00	29.00	29.00	38.45	32.00	34.55	43.00
70–85	18.00	19.00	28.90	23.00	23.00	34.35	26.00	28.00	40.00	29.00	31.00	44.00
	3.5			3.6			3.7			3.8		
	p1	p5	p95	p1	p5	p95	p1	p5	p95	p1	p5	p95
18–29	34.00	35.55	46.90	27.00	29.65	39.45	19.00	23.00	31.00	16.00	16.00	26.00

30–39	31.00	34.00	45.90	27.00	28.00	41.45	21.00	21.55	31.45	14.00	15.00	26.00
40–49	37.00	38.55	45.45	32.00	32.00	41.70	23.00	24.55	34.45	16.00	16.55	27.45
50–59	34.00	36.00	47.35	29.00	29.55	42.00	21.00	22.55	32.00	13.00	13.00	26.45
60–69	31.00	33.55	43.00	25.00	27.55	37.45	18.00	21.10	29.00	13.00	14.55	23.45
70–85	29.00	30.10	46.45	24.00	25.65	42.90	19.00	20.55	34.45	14.00	14.55	25.00
		4.1			4.2			4.3			4.4	
	p1	p5	p95	p1	p5	p95	p1	p5	p95	p1	p5	p95
18–29	19.00	22.55	32.90	28.00	28.00	41.00	38.00	38.00	48.00	27.00	28.55	41.00
30–39	22.00	23.55	33.80	29.00	30.00	39.45	37.00	38.10	48.00	28.00	29.55	42.45
40–49	22.00	23.55	33.90	30.00	31.55	40.45	38.00	40.00	49.90	22.00	27.00	42.90
50–59	23.00	24.00	33.45	31.00	32.00	40.45	38.00	38.00	48.45	29.00	30.55	42.00
60–69	21.00	22.00	32.00	28.00	29.10	39.00	36.00	37.00	45.45	26.00	27.00	40.00
70–85	19.00	22.55	34.45	26.00	27.55	39.45	32.00	34.00	48.35	26.00	26.55	41.45
		4.5			4.6			4.7			4.8	
	p1	p5	p95	p1	p5	p95	p1	p5	p95	p1	p5	p95
18–29	27.00	28.65	43.90	38.00	39.10	50.00	28.00	29.55	37.45	21.00	21.00	31.00
30–39	28.00	29.00	41.35	37.00	38.00	50.00	26.00	28.55	39.00	19.00	19.55	32.90
40–49	26.00	27.55	42.00	37.00	39.20	49.45	30.00	30.55	42.80	20.00	21.00	35.45
50–59	28.00	29.00	44.00	40.00	40.00	50.00	28.00	30.00	39.90	18.00	20.00	31.00
60–69	27.00	27.00	39.45	34.00	36.00	46.45	24.00	27.00	36.00	17.00	18.55	27.00
70–85	25.00	26.65	41.80	33.00	34.00	47.90	23.00	24.00	39.45	16.00	17.55	29.00
**IPL**		5.1			5.2			5.3			5.4	
	p1	p5	p95	p1	p5	p95	p1	p5	p95	p1	p5	p95
18–29	24.00	26.00	36.00	29.00	31.55	43.00	36.00	37.55	48.45	29.00	29.55	40.90
30–39	23.00	25.55	35.45	30.00	31.55	42.00	36.00	37.10	49.00	24.00	25.55	39.45
40–49	23.00	24.55	37.90	29.00	32.55	43.00	36.00	37.55	50.00	25.00	26.65	41.90
50–59	23.00	26.10	37.45	30.00	32.55	43.00	38.00	40.00	49.00	25.00	26.55	40.45
60–69	24.00	25.55	34.00	29.00	30.00	40.45	35.00	36.55	46.45	24.00	25.00	37.45
70–85	22.00	23.55	36.45	28.00	28.55	43.45	32.00	34.55	49.00	19.00	22.10	41.80
		5.5			5.6			5.7			5.8	
	p1	p5	p95	p1	p5	p95	p1	p5	p95	p1	p5	p95
18–29	31.00	31.00	44.00	36.00	38.55	47.00	27.00	27.55	36.00	18.00	20.00	29.00
30–39	28.00	28.55	41.35	35.00	36.10	48.45	24.00	25.10	38.00	17.00	18.55	28.45
40–49	24.00	30.00	45.00	37.00	37.55	49.00	27.00	28.00	39.00	17.00	17.00	31.00
50–59	29.00	30.55	44.00	38.00	39.00	49.00	28.00	29.00	39.00	18.00	19.00	30.90
60–69	28.00	28.00	39.00	33.00	35.55	46.00	26.00	26.00	36.00	18.00	18.00	27.90
70–85	23.00	26.20	44.45	28.00	33.00	46.90	23.00	24.10	38.35	16.00	17.00	28.90
		6.1			6.2			6.3			6.4	
	p1	p5	p95	p1	p5	p95	p1	p5	p95	p1	p5	p95
18–29	20.00	22.55	31.45	24.00	26.10	35.45	29.00	29.55	41.45	35.00	36.00	46.00
30–39	20.00	20.55	32.35	24.00	25.55	37.00	27.00	29.55	40.45	35.00	35.00	45.45
40–49	19.00	23.00	33.45	25.00	27.00	36.90	29.00	30.55	42.80	33.00	35.10	47.35
50–59	19.00	21.00	32.45	25.00	26.00	37.45	31.00	32.55	42.45	37.00	37.00	48.00
60–69	21.00	22.10	30.00	25.00	25.55	35.00	30.00	30.00	38.00	35.00	35.00	44.00
70–85	20.00	20.00	32.00	23.00	25.00	35.00	25.00	28.00	39.45	27.00	31.10	43.45
		6.5			6.6			6.7			6.8	
	p1	p5	p95	p1	p5	p95	p1	p5	p95	p1	p5	p95
18–29	34.00	36.00	45.45	28.00	29.00	38.00	22.00	22.55	31.45	15.00	16.55	24.45
30–39	35.00	36.00	47.00	26.00	28.55	40.00	21.00	22.00	33.45	15.00	16.00	26.00
40–49	33.00	35.55	47.80	24.00	29.55	43.45	20.00	22.55	33.80	14.00	16.00	26.45
50–59	36.00	37.00	49.00	31.00	31.00	40.45	23.00	24.00	33.00	16.00	17.00	27.00
60–69	34.00	35.00	44.90	26.00	27.00	37.90	21.00	21.00	29.90	15.00	16.55	25.00
70–85	25.00	30.00	44.90	24.00	26.10	39.35	20.00	21.55	30.00	15.00	15.55	24.90
		7.1			7.2			7.3			7.4	
	p1	p5	p95	p1	p5	p95	p1	p5	p95	p1	p5	p95
18–29	17.00	18.00	25.00	19.00	20.00	28.45	23.00	23.00	31.45	26.00	26.55	35.45
30–39	15.00	15.55	26.00	18.00	19.00	27.90	20.00	21.00	32.45	23.00	25.00	36.00
40–49	17.00	17.00	26.00	19.00	20.00	30.00	22.00	23.00	33.00	24.00	26.55	36.90
50–59	14.00	15.55	25.90	16.00	18.55	29.00	22.00	22.00	33.45	25.00	27.00	36.45
60–69	16.00	16.55	23.45	19.00	20.00	27.00	22.00	23.00	30.00	25.00	26.00	34.45
70–85	14.00	15.00	25.45	17.00	18.10	29.45	20.00	21.00	32.00	21.00	22.00	34.45
		7.5			7.6			7.7			7.8	
	p1	p5	p95	p1	p5	p95	p1	p5	p95	p1	p5	p95

18–29	25.00	26.55	35.45	20.00	21.55	31.00	17.00	18.00	26.45	13.00	14.55	23.45
30–39	24.00	25.00	36.00	20.00	21.00	30.45	16.00	16.55	26.00	13.00	14.00	22.00
40–49	26.00	27.00	35.35	21.00	21.55	31.00	16.00	17.00	27.00	11.00	14.00	22.45
50–59	25.00	27.00	38.00	21.00	21.55	33.00	17.00	17.00	27.00	12.00	15.00	23.90
60–69	23.00	24.65	33.00	19.00	20.55	29.00	14.00	17.55	24.45	12.00	13.00	21.00
70–85	21.00	21.00	34.00	17.00	19.00	31.00	16.00	16.00	24.45	11.00	14.10	21.45
		8.1			8.2			8.3			8.4	
	p1	p5	p95	p1	p5	p95	p1	p5	p95	p1	p5	p95
18–29	13.00	14.00	20.45	15.00	16.00	23.00	16.00	18.00	25.45	20.00	20.00	27.90
30–39	12.00	13.00	21.45	14.00	14.55	24.00	15.00	16.00	26.45	16.00	18.00	28.35
40–49	13.00	14.00	22.90	15.00	16.00	25.45	16.00	16.55	27.00	18.00	18.55	28.45
50–59	12.00	12.55	21.00	13.00	15.55	23.00	15.00	17.00	26.90	18.00	19.55	28.00
60–69	12.00	14.00	21.00	15.00	15.00	23.45	17.00	17.00	24.00	17.00	19.00	27.00
70–85	12.00	12.00	22.45	14.00	14.00	24.45	15.00	16.00	27.00	18.00	18.00	28.00
		8.5			8.6			8.7			8.8	
	p1	p5	p95	p1	p5	p95	p1	p5	p95	p1	p5	p95
18–29	20.00	21.00	28.00	17.00	18.00	26.00	14.00	14.55	24.45	10.00	12.10	22.90
30–39	18.00	19.10	29.90	16.00	16.55	25.90	14.00	14.00	22.00	11.00	12.55	22.45
40–49	18.00	19.00	28.90	16.00	16.00	26.00	13.00	14.00	23.45	10.00	12.10	23.45
50–59	20.00	21.00	29.45	16.00	17.55	26.45	14.00	15.00	23.00	12.00	13.55	26.90
60–69	19.00	20.00	27.45	15.00	16.55	25.45	14.00	14.55	23.45	12.00	12.55	23.90
70–85	17.00	17.55	29.00	14.00	16.55	25.45	14.00	14.55	24.00	11.00	11.55	24.00

Table S4. 1st, 5th and 95th percentiles values of the inner nuclear layer (INL) thickness in each of the 64 cells of the 8 × 8 macular grid in the different age groups.

INL		**1.1**			**1.2**			**1.3**			**1.4**	
	**p1**	**p5**	**p95**	**p1**	**p5**	**p95**	**p1**	**p5**	**p95**	**p1**	**p5**	**p95**
18–29	19.00	21.55	30.45	21.00	22.00	30.00	21.00	23.10	32.45	21.00	24.00	31.35
30–39	19.00	21.00	32.25	20.00	22.00	30.45	22.00	22.00	30.00	22.00	22.55	34.25
40–49	20.00	21.00	28.00	22.00	22.00	30.00	22.00	23.00	31.00	20.00	22.10	31.80
50–59	20.00	20.55	29.90	20.00	21.55	28.00	20.00	22.00	29.45	21.00	22.55	30.00
60–69	17.00	19.00	38.45	20.00	21.00	34.45	21.00	22.00	34.35	22.00	22.00	37.80
70–85	19.00	20.00	34.90	20.00	20.00	30.90	20.00	20.55	34.00	21.00	22.00	37.25
		1.5			1.6			1.7			1.8	
	p1	p5	p95	p1	p5	p95	p1	p5	p95	p1	p5	p95
18–29	21.00	23.00	32.35	21.00	22.00	32.35	21.00	21.55	31.45	21.00	21.00	33.80
30–39	22.00	23.00	33.25	21.00	21.00	31.00	20.00	20.55	32.90	19.00	20.00	32.80
40–49	21.00	23.00	32.00	21.00	22.00	31.00	19.00	20.55	32.45	18.00	20.55	31.45
50–59	20.00	22.10	31.45	21.00	22.00	33.45	20.00	21.00	36.00	21.00	21.55	34.80
60–69	22.00	22.00	37.45	21.00	22.00	33.00	20.00	21.00	34.90	17.00	19.55	40.25
70–85	21.00	21.55	35.45	20.00	22.10	36.35	20.00	20.00	34.90	18.00	18.55	38.90
		2.1			2.2			2.3			2.4	
	p1	p5	p95	p1	p5	p95	p1	p5	p95	p1	p5	p95
18–29	23.00	23.55	30.45	24.00	25.00	32.00	26.00	27.00	35.00	24.00	29.00	38.45
30–39	22.00	22.00	31.00	22.00	23.55	31.45	25.00	25.55	34.00	25.00	27.55	36.45
40–49	22.00	23.00	30.45	23.00	23.55	31.35	26.00	27.00	35.00	27.00	29.00	36.90
50–59	22.00	22.55	29.45	21.00	23.55	31.00	24.00	24.55	34.90	28.00	28.55	37.00
60–69	19.00	21.55	29.45	22.00	23.00	34.05	25.00	26.00	40.85	27.00	27.00	41.15
70–85	20.00	20.00	30.90	21.00	21.00	31.00	25.00	25.00	35.00	26.00	27.00	41.45
		2.5			2.6			2.7			2.8	
	p1	p5	p95	p1	p5	p95	p1	p5	p95	p1	p5	p95
18–29	25.00	27.00	40.00	24.00	28.00	38.45	24.00	24.55	35.45	21.00	22.00	34.45
30–39	26.00	27.55	36.45	26.00	26.55	37.00	23.00	23.55	33.45	20.00	22.00	32.45
40–49	28.00	29.10	37.00	28.00	28.55	36.35	25.00	25.55	34.45	18.00	20.55	31.00
50–59	27.00	27.55	38.00	26.00	26.55	36.00	22.00	22.55	32.90	21.00	22.00	32.90
60–69	27.00	27.00	42.90	25.00	25.55	37.80	22.00	23.55	35.00	20.00	20.55	33.90
70–85	26.00	26.55	41.45	25.00	26.00	38.90	22.00	23.10	33.90	20.00	20.55	34.00
		3.1			3.2			3.3			3.4	
	p1	p5	p95	p1	p5	p95	p1	p5	p95	p1	p5	p95
18–29	25.00	25.55	32.00	27.00	28.00	37.45	32.00	32.55	42.45	33.00	35.00	48.35
30–39	24.00	24.55	34.00	27.00	27.55	35.45	32.00	32.55	41.90	35.00	35.55	45.45
40–49	24.00	25.00	32.90	25.00	29.00	38.45	33.00	33.55	42.00	35.00	36.00	46.45

50–59	22.00	25.00	32.45	27.00	28.00	39.00	33.00	34.00	43.90	35.00	36.00	46.45
60–69	21.00	22.00	31.00	25.00	26.55	35.45	32.00	32.00	44.80	35.00	35.00	48.00
70–85	21.00	21.55	32.45	26.00	26.55	37.00	28.00	30.00	41.45	30.00	31.65	45.45
		3.5			3.6			3.7			3.8	
	p1	p5	p95	p1	p5	p95	p1	p5	p95	p1	p5	p95
18–29	35.00	35.55	48.90	32.00	34.00	47.25	28.00	28.55	40.00	23.00	25.55	35.45
30–39	35.00	36.00	47.00	32.00	33.00	44.00	27.00	27.00	38.45	24.00	24.55	37.45
40–49	35.00	37.00	48.45	34.00	34.00	47.35	29.00	30.00	39.90	23.00	24.65	36.00
50–59	35.00	36.00	50.00	32.00	32.55	45.00	28.00	29.00	38.00	22.00	22.55	34.45
60–69	33.00	36.00	47.45	32.00	32.55	43.00	27.00	27.00	34.00	23.00	24.00	32.45
70–85	33.00	34.00	45.00	30.00	31.00	47.00	22.00	26.00	39.90	23.00	23.00	35.90
		4.1			4.2			4.3			4.4	
	p1	p5	p95	p1	p5	p95	p1	p5	p95	p1	p5	p95
18–29	25.00	26.55	35.45	31.00	32.55	40.45	34.00	35.00	45.45	24.00	26.00	41.90
30–39	25.00	26.55	35.00	30.00	31.55	40.45	33.00	34.55	45.45	25.00	26.00	41.45
40–49	26.00	26.55	35.45	30.00	32.55	42.00	36.00	36.55	47.00	21.00	26.55	42.45
50–59	22.00	25.00	34.45	27.00	30.00	40.45	32.00	35.00	47.00	26.00	29.00	42.45
60–69	23.00	24.55	33.45	29.00	30.55	42.00	32.00	33.00	50.35	25.00	26.00	42.00
70–85	22.00	23.55	37.00	27.00	28.00	39.45	29.00	31.55	46.00	24.00	24.55	46.35
		4.5			4.6			4.7			4.8	
	p1	p5	p95	p1	p5	p95	p1	p5	p95	p1	p5	p95
18–29	27.00	28.00	43.00	36.00	37.00	53.70	31.00	32.00	43.45	27.00	29.00	38.35
30–39	25.00	27.55	42.00	35.00	37.10	52.45	29.00	31.10	42.90	26.00	26.55	39.00
40–49	23.00	25.55	46.90	31.00	36.55	54.00	33.00	33.00	48.90	25.00	26.00	41.00
50–59	24.00	27.20	43.45	36.00	37.55	54.45	31.00	31.55	44.35	25.00	25.00	37.45
60–69	23.00	29.00	45.35	33.00	36.00	50.35	30.00	31.55	41.45	25.00	25.55	39.00
70–85	23.00	27.10	48.00	34.00	35.55	56.00	29.00	29.55	48.00	22.00	23.55	38.45
INL		5.1			5.2			5.3			5.4	
	p1	p5	p95	p1	p5	p95	p1	p5	p95	p1	p5	p95
18–29	25.00	25.55	34.45	29.00	30.00	39.45	33.00	34.00	44.45	24.00	25.55	40.00
30–39	25.00	25.00	33.45	27.00	29.00	41.00	31.00	34.55	46.00	21.00	24.00	37.90
40–49	24.00	26.55	35.45	28.00	29.00	40.00	33.00	36.00	45.00	25.00	26.10	41.00
50–59	23.00	24.00	34.00	27.00	29.00	39.45	34.00	36.00	46.90	24.00	26.10	39.45
60–69	22.00	24.00	32.45	27.00	28.55	37.90	31.00	32.55	45.45	20.00	23.10	39.45
70–85	21.00	23.55	32.90	24.00	26.55	38.45	30.00	30.55	45.45	20.00	24.65	42.45
		5.5			5.6			5.7			5.8	
	p1	p5	p95	p1	p5	p95	p1	p5	p95	p1	p5	p95
18–29	27.00	28.55	41.45	35.00	35.55	48.45	32.00	33.00	41.90	25.00	25.55	38.00
30–39	25.00	27.55	41.00	37.00	37.55	49.90	30.00	31.55	41.45	24.00	26.00	37.45
40–49	28.00	28.00	45.90	39.00	39.55	53.45	31.00	32.00	43.45	22.00	26.55	38.90
50–59	27.00	28.00	44.45	36.00	37.55	54.00	32.00	32.00	42.45	22.00	26.00	40.00
60–69	27.00	28.55	44.70	33.00	37.00	52.25	32.00	32.00	41.45	26.00	26.00	36.00
70–85	25.00	28.00	49.45	32.00	34.55	53.00	28.00	28.55	46.45	24.00	24.00	39.80
		6.1			6.2			6.3			6.4	
	p1	p5	p95	p1	p5	p95	p1	p5	p95	p1	p5	p95
18–29	24.00	24.55	33.00	28.00	29.00	37.00	33.00	33.55	43.45	33.00	34.55	45.45
30–39	24.00	25.55	32.90	27.00	27.55	38.45	32.00	34.00	44.45	34.00	34.55	45.45
40–49	22.00	25.10	34.45	28.00	29.00	39.00	33.00	33.55	45.45	34.00	35.55	49.90
50–59	22.00	23.55	34.00	24.00	27.00	37.45	32.00	33.00	43.45	34.00	35.55	47.25
60–69	22.00	23.55	33.45	26.00	27.00	37.00	30.00	31.00	42.45	33.00	33.55	46.00
70–85	23.00	23.55	33.00	23.00	27.00	38.45	29.00	29.55	42.45	30.00	32.00	45.00
		6.5			6.6			6.7			6.8	
	p1	p5	p95	p1	p5	p95	p1	p5	p95	p1	p5	p95
18–29	32.00	34.55	46.00	33.00	33.00	45.00	28.00	28.55	37.45	25.00	26.00	36.45
30–39	34.00	35.55	45.45	33.00	34.00	43.00	27.00	27.55	37.45	25.00	25.00	35.45
40–49	36.00	36.00	48.00	34.00	34.55	44.45	26.00	28.55	38.45	24.00	24.55	36.00
50–59	35.00	37.00	48.45	33.00	34.55	43.90	29.00	29.00	36.45	23.00	24.55	34.00
60–69	32.00	34.55	46.45	31.00	32.55	42.90	27.00	28.55	36.00	23.00	24.00	34.45
70–85	30.00	31.55	46.45	26.00	30.55	44.80	25.00	26.65	38.00	23.00	23.00	33.45
		7.1			7.2			7.3			7.4	
	p1	p5	p95	p1	p5	p95	p1	p5	p95	p1	p5	p95
18–29	23.00	24.00	32.00	26.00	26.00	33.00	27.00	27.55	36.45	27.00	27.55	37.90
30–39	23.00	23.00	30.00	24.00	25.00	32.45	25.00	27.00	35.45	27.00	27.00	36.45

40–49	23.00	24.00	31.45	25.00	25.00	34.45	25.00	26.55	36.90	27.00	27.55	37.45
50–59	20.00	21.00	32.00	21.00	23.10	36.00	25.00	26.55	35.45	27.00	27.55	37.00
60–69	20.00	21.55	29.45	23.00	23.55	31.45	24.00	26.55	35.45	26.00	26.55	37.00
70–85	21.00	21.55	30.90	22.00	22.55	32.90	23.00	25.00	35.45	26.00	26.00	37.45
		7.5			7.6			7.7			7.8	
	p1	p5	p95	p1	p5	p95	p1	p5	p95	p1	p5	p95
18–29	28.00	28.00	39.00	27.00	27.55	38.00	25.00	26.00	33.45	24.00	25.00	34.00
30–39	26.00	26.55	35.00	25.00	26.55	35.00	24.00	24.55	33.45	22.00	24.00	34.90
40–49	27.00	28.55	38.00	26.00	27.55	36.45	25.00	25.55	34.90	23.00	24.00	31.45
50–59	26.00	29.00	37.00	27.00	27.00	35.45	24.00	24.55	35.00	23.00	23.00	33.45
60–69	28.00	28.55	37.45	26.00	27.00	36.45	23.00	23.55	32.00	22.00	22.55	32.45
70–85	23.00	26.55	37.45	22.00	24.55	37.35	23.00	24.00	33.45	22.00	23.00	32.00
		8.1			8.2			8.3			8.4	
	p1	p5	p95	p1	p5	p95	p1	p5	p95	p1	p5	p95
18–29	22.00	22.00	30.00	22.00	23.00	30.45	22.00	23.00	31.45	22.00	23.55	32.45
30–39	21.00	22.00	27.00	20.00	22.00	29.00	21.00	23.00	30.00	22.00	23.00	31.00
40–49	22.00	22.55	30.90	23.00	23.00	32.45	23.00	23.55	33.35	24.00	24.55	34.35
50–59	21.00	21.55	31.45	19.00	21.55	29.00	22.00	22.55	30.45	22.00	22.55	31.00
60–69	20.00	20.00	27.00	21.00	21.55	28.00	21.00	22.00	31.45	21.00	22.55	30.45
70–85	18.00	18.55	29.00	19.00	20.00	28.45	21.00	21.55	30.00	21.00	22.00	32.45
		8.5			8.6			8.7			8.8	
	p1	p5	p95	p1	p5	p95	p1	p5	p95	p1	p5	p95
18–29	23.00	23.55	31.45	22.00	23.00	33.00	23.00	24.00	31.00	22.00	23.00	32.00
30–39	23.00	23.55	31.00	23.00	23.00	31.00	22.00	23.00	31.45	23.00	23.00	31.00
40–49	23.00	23.55	32.35	23.00	24.00	31.45	22.00	23.00	31.00	22.00	23.00	31.45
50–59	23.00	23.00	32.00	22.00	23.55	32.45	22.00	23.00	30.45	21.00	23.00	32.45
60–69	22.00	22.55	32.45	21.00	22.00	32.00	21.00	22.00	32.35	22.00	22.00	37.45
70–85	21.00	22.55	33.45	21.00	22.55	34.00	21.00	22.00	35.80	20.00	20.55	37.25

Table S5. 1st, 5th and 95th percentiles values of the outer plexiform layer (OPL) thickness in each of the 64 cells of the 8 × 8 macular grid in the different age groups.

**OPL**		**1.1**			**1.2**			**1.3**			**1.4**	
	**p1**	**p5**	**p95**	**p1**	**p5**	**p95**	**p1**	**p5**	**p95**	**p1**	**p5**	**p95**
18–29	19.00	20.00	25.45	19.00	20.00	25.00	19.00	20.55	25.45	21.00	21.00	27.00
30–39	18.00	20.00	25.00	20.00	20.00	25.45	21.00	21.00	25.45	21.00	21.55	27.00
40–49	20.00	20.00	23.45	19.00	20.00	25.00	21.00	21.00	25.45	21.00	21.00	27.00
50–59	19.00	20.00	25.00	20.00	20.00	25.00	21.00	21.00	25.90	20.00	20.55	27.45
60–69	19.00	19.55	24.90	19.00	19.00	25.90	20.00	21.00	27.35	20.00	20.55	27.45
70–85	18.00	19.00	27.70	18.00	19.55	25.45	18.00	20.00	26.00	19.00	20.55	26.45
		1.5			1.6			1.7			1.8	
	p1	p5	p95	p1	p5	p95	p1	p5	p95	p1	p5	p95
18–29	21.00	21.55	28.00	20.00	20.55	27.00	20.00	20.00	26.00	19.00	20.00	25.45
30–39	20.00	21.00	27.45	20.00	20.55	26.00	18.00	19.55	25.00	19.00	20.00	25.45
40–49	21.00	21.55	28.00	20.00	21.00	28.00	18.00	19.00	27.00	19.00	20.00	26.00
50–59	21.00	21.00	28.00	19.00	20.00	28.45	19.00	19.00	26.00	18.00	20.00	28.00
60–69	20.00	20.00	27.45	18.00	20.00	25.90	19.00	19.00	26.00	18.00	19.00	27.45
70–85	20.00	20.55	27.90	20.00	20.00	26.45	19.00	19.55	26.00	19.00	19.00	28.45
		2.1			2.2			2.3			2.4	
	p1	p5	p95	p1	p5	p95	p1	p5	p95	p1	p5	p95
18–29	20.00	20.00	26.00	20.00	21.00	26.45	22.00	22.00	29.45	23.00	24.00	34.00
30–39	19.00	20.10	25.00	20.00	21.00	27.00	22.00	22.00	29.45	22.00	23.00	31.90
40–49	20.00	20.00	25.00	20.00	21.00	26.45	21.00	22.55	28.45	22.00	23.55	32.25
50–59	20.00	21.00	25.00	21.00	21.00	28.35	22.00	22.00	29.00	22.00	23.00	33.00
60–69	19.00	20.55	26.35	20.00	21.00	29.00	21.00	21.55	33.80	22.00	22.55	31.45
70–85	19.00	20.55	25.45	20.00	21.00	27.00	20.00	22.00	28.45	22.00	23.00	32.45
		2.5			2.6			2.7			2.8	
	p1	p5	p95	p1	p5	p95	p1	p5	p95	p1	p5	p95
18–29	23.00	24.00	35.25	22.00	22.55	32.45	20.00	21.55	29.00	20.00	20.00	27.00
30–39	23.00	23.00	33.45	22.00	22.00	32.00	20.00	21.00	29.00	19.00	19.55	25.45
40–49	22.00	23.55	39.45	21.00	22.55	35.45	21.00	22.00	31.45	19.00	20.00	28.90
50–59	22.00	23.00	36.35	21.00	22.00	33.45	20.00	21.00	29.00	19.00	19.00	27.90
60–69	20.00	22.55	32.45	21.00	22.00	32.00	20.00	20.55	28.00	18.00	19.55	26.00

70–85	22.00	23.00	35.00	21.00	22.00	33.00	20.00	21.00	30.45	19.00	20.00	26.00
		3.1			3.2			3.3			3.4	
	p1	p5	p95	p1	p5	p95	p1	p5	p95	p1	p5	p95
18–29	20.00	20.55	28.00	22.00	22.00	30.45	24.00	24.55	36.45	25.00	26.00	49.90
30–39	19.00	21.55	28.00	22.00	23.00	30.45	24.00	25.00	34.00	26.00	26.55	45.00
40–49	21.00	21.55	27.00	22.00	22.00	29.45	23.00	24.00	34.00	24.00	25.55	43.35
50–59	19.00	20.00	28.45	23.00	23.00	32.80	24.00	24.00	37.25	25.00	26.00	46.80
60–69	20.00	21.00	28.45	21.00	22.00	34.45	23.00	23.55	40.00	24.00	25.55	46.50
70–85	20.00	20.55	28.00	21.00	22.00	31.00	23.00	24.00	36.00	26.00	27.00	49.45
		3.5			3.6			3.7			3.8	
	p1	p5	p95	p1	p5	p95	p1	p5	p95	p1	p5	p95
18–29	26.00	27.00	53.45	23.00	25.00	40.45	23.00	23.00	34.00	22.00	22.00	30.45
30–39	26.00	27.00	44.80	25.00	25.00	40.00	23.00	23.00	35.45	21.00	21.00	29.45
40–49	26.00	26.55	53.80	24.00	25.55	46.45	23.00	23.55	36.00	21.00	22.00	31.00
50–59	25.00	25.00	47.00	24.00	25.00	46.00	24.00	24.00	37.45	21.00	21.55	31.45
60–69	26.00	26.00	50.45	24.00	25.00	43.00	23.00	24.00	34.90	21.00	21.00	28.00
70–85	25.00	26.55	54.45	24.00	24.55	49.90	24.00	24.00	36.00	22.00	22.00	28.45
		4.1			4.2			4.3			4.4	
	p1	p5	p95	p1	p5	p95	p1	p5	p95	p1	p5	p95
18–29	20.00	21.55	30.90	22.00	23.00	33.45	25.00	25.55	44.90	22.00	22.55	59.45
30–39	21.00	22.55	28.45	24.00	24.00	33.80	26.00	26.00	45.80	23.00	24.00	49.90
40–49	21.00	22.00	29.45	23.00	24.00	34.00	25.00	25.00	41.80	18.00	23.55	49.15
50–59	19.00	22.00	32.35	23.00	24.00	40.25	25.00	25.00	51.25	21.00	23.00	53.90
60–69	19.00	21.00	32.35	21.00	23.00	41.80	24.00	25.00	53.90	21.00	23.00	58.45
70–85	21.00	21.55	30.00	23.00	23.00	34.45	25.00	25.00	45.15	23.00	24.00	50.90
		4.5			4.6			4.7			4.8	
	p1	p5	p95	p1	p5	p95	p1	p5	p95	p1	p5	p95
18–29	22.00	23.55	61.90	26.00	27.00	51.90	24.00	25.00	37.80	22.00	24.00	33.45
30–39	24.00	24.55	52.45	25.00	26.10	58.70	24.00	25.55	45.45	23.00	23.55	35.45
40–49	24.00	25.55	59.35	25.00	26.00	61.25	24.00	25.55	45.70	22.00	23.55	35.45
50–59	22.00	24.00	54.45	26.00	26.55	56.00	24.00	24.55	42.45	23.00	23.00	35.00
60–69	24.00	25.00	56.90	25.00	26.55	62.70	25.00	26.00	42.00	23.00	24.00	33.00
70–85	21.00	24.10	63.45	26.00	27.10	63.15	23.00	25.55	49.70	22.00	22.00	33.00
**OPL**		5.1			5.2			5.3			5.4	
	p1	p5	p95	p1	p5	p95	p1	p5	p95	p1	p5	p95
18–29	21.00	21.00	31.45	23.00	24.00	37.00	24.00	25.00	41.00	21.00	22.00	48.00
30–39	21.00	21.55	29.90	21.00	24.55	33.00	24.00	25.55	39.00	22.00	23.00	41.90
40–49	21.00	22.55	29.00	24.00	24.00	34.35	24.00	25.00	41.45	22.00	23.00	52.50
50–59	20.00	21.00	31.90	23.00	24.00	36.80	23.00	24.55	45.15	22.00	23.00	47.90
60–69	20.00	21.00	31.45	22.00	23.10	37.90	24.00	24.55	48.05	22.00	22.00	44.70
70–85	22.00	22.55	30.45	24.00	24.00	33.00	25.00	26.00	35.45	19.00	22.00	41.25
		5.5			5.6			5.7			5.8	
	p1	p5	p95	p1	p5	p95	p1	p5	p95	p1	p5	p95
18–29	21.00	22.00	48.00	24.00	25.00	47.90	24.00	24.55	35.00	21.00	22.00	31.45
30–39	22.00	22.55	54.45	24.00	26.00	56.25	25.00	25.00	43.45	22.00	22.55	35.45
40–49	25.00	25.00	59.90	25.00	26.00	56.00	24.00	25.00	46.70	22.00	23.00	33.45
50–59	21.00	22.55	57.90	25.00	26.00	53.35	25.00	25.00	41.00	22.00	23.00	31.45
60–69	22.00	23.00	58.70	25.00	26.00	59.35	24.00	25.00	43.90	21.00	22.00	30.90
70–85	23.00	23.00	58.70	26.00	27.00	57.90	23.00	25.00	41.45	22.00	23.00	34.45
		6.1			6.2			6.3			6.4	
	p1	p5	p95	p1	p5	p95	p1	p5	p95	p1	p5	p95
18–29	19.00	20.00	28.90	22.00	22.00	31.45	22.00	23.55	34.00	24.00	25.00	41.00
30–39	21.00	21.00	28.45	23.00	23.55	32.45	25.00	25.00	34.45	26.00	26.00	36.90
40–49	21.00	21.00	28.00	21.00	22.55	30.90	24.00	24.55	34.35	24.00	25.00	40.25
50–59	19.00	19.55	30.90	21.00	22.55	34.00	23.00	24.55	33.90	24.00	25.00	36.90
60–69	20.00	21.00	29.00	21.00	22.55	33.90	23.00	23.55	34.00	23.00	24.00	40.45
70–85	20.00	21.10	28.00	22.00	23.00	30.00	24.00	24.00	36.00	24.00	24.55	44.25
		6.5			6.6			6.7			6.8	
	p1	p5	p95	p1	p5	p95	p1	p5	p95	p1	p5	p95
18–29	25.00	25.00	37.90	23.00	24.00	33.90	21.00	22.00	30.00	18.00	21.00	27.00
30–39	25.00	25.55	39.80	24.00	24.00	36.90	22.00	22.00	33.00	21.00	21.55	30.00
40–49	26.00	26.00	44.70	24.00	25.00	41.80	20.00	22.55	36.00	15.00	21.00	29.45
50–59	25.00	25.55	37.45	22.00	24.55	37.90	23.00	23.00	31.90	21.00	21.55	27.45

60–69	24.00	25.55	42.00	23.00	24.00	44.90	22.00	22.55	36.45	21.00	21.55	29.45
70–85	24.00	25.00	58.95	22.00	24.00	49.25	21.00	22.00	37.00	21.00	22.00	29.45
	7.1			7.2			7.3			7.4		
	p1	p5	p95	p1	p5	p95	p1	p5	p95	p1	p5	p95
18–29	20.00	20.55	26.00	21.00	21.55	27.45	22.00	22.00	30.00	22.00	23.00	32.35
30–39	20.00	21.00	26.45	21.00	22.55	27.45	22.00	23.00	29.45	23.00	23.00	31.90
40–49	20.00	20.00	26.45	20.00	22.00	27.45	22.00	23.00	31.45	22.00	22.55	32.35
50–59	19.00	19.55	27.00	21.00	22.00	29.90	21.00	22.00	30.90	21.00	22.00	30.00
60–69	20.00	21.00	27.00	20.00	21.00	27.45	21.00	21.55	29.45	22.00	23.00	30.45
70–85	19.00	19.55	26.00	20.00	21.00	27.00	21.00	22.00	28.90	20.00	22.00	31.00
	7.5			7.6			7.7			7.8		
	p1	p5	p95	p1	p5	p95	p1	p5	p95	p1	p5	p95
18–29	22.00	22.55	30.45	21.00	21.00	28.45	21.00	21.00	26.00	20.00	20.55	25.90
30–39	23.00	23.00	32.35	22.00	22.00	30.45	21.00	22.00	28.00	20.00	20.00	27.00
40–49	22.00	23.00	33.25	21.00	22.00	31.80	22.00	22.00	30.45	19.00	20.00	29.00
50–59	22.00	22.55	30.35	21.00	21.55	29.45	20.00	21.00	28.00	21.00	21.00	26.00
60–69	22.00	23.00	32.00	20.00	22.00	30.45	20.00	20.55	28.90	19.00	20.00	26.45
70–85	19.00	21.55	37.00	19.00	21.55	33.00	20.00	21.00	29.00	21.00	21.00	26.45
	8.1			8.2			8.3			8.4		
	p1	p5	p95	p1	p5	p95	p1	p5	p95	p1	p5	p95
18–29	18.00	19.55	25.00	19.00	20.00	25.00	20.00	21.00	26.45	20.00	21.00	28.00
30–39	20.00	21.00	26.00	21.00	21.00	25.00	21.00	21.00	26.45	21.00	21.00	26.45
40–49	19.00	19.55	26.00	20.00	20.55	26.45	21.00	21.00	28.90	21.00	21.55	29.35
50–59	17.00	17.55	26.00	18.00	20.00	25.45	21.00	21.00	26.90	20.00	21.00	27.45
60–69	20.00	20.00	25.45	20.00	20.55	25.45	19.00	20.55	26.00	20.00	20.00	27.00
70–85	19.00	20.00	25.00	20.00	20.00	26.00	20.00	20.55	25.00	20.00	21.00	27.00
	8.5			8.6			8.7			8.8		
	p1	p5	p95	p1	p5	p95	p1	p5	p95	p1	p5	p95
18–29	21.00	21.00	26.45	20.00	20.00	25.45	20.00	20.00	25.00	19.00	20.00	24.45
30–39	20.00	21.00	27.45	20.00	20.55	27.00	21.00	21.00	25.45	18.00	19.55	26.00
40–49	21.00	22.00	28.35	20.00	21.00	26.90	20.00	20.55	26.45	19.00	20.00	25.45
50–59	20.00	21.00	26.00	20.00	20.55	27.00	20.00	20.55	24.45	18.00	19.00	25.00
60–69	20.00	20.55	27.35	19.00	20.55	26.45	19.00	19.55	25.45	19.00	19.55	24.45
70–85	20.00	20.00	26.00	19.00	20.55	27.00	19.00	20.00	27.00	19.00	19.55	30.45

Table S6. 1st, 5th and 95th percentiles values of the outer nuclear layer (ONL) thickness in each of the 64 cells of the 8 × 8 macular grid in the different age groups.

ONL	**1.1**			**1.2**			**1.3**			**1.4**		
	**p1**	**p5**	**p95**	**p1**	**p5**	**p95**	**p1**	**p5**	**p95**	**p1**	**p5**	**p95**
18–29	35.00	39.55	58.45	37.00	38.10	56.45	37.00	38.55	57.90	37.00	39.00	58.45
30–39	40.00	41.00	54.00	40.00	40.55	58.00	39.00	39.55	59.45	37.00	39.00	58.45
40–49	23.00	37.00	55.00	26.00	37.55	55.90	26.00	38.00	56.45	26.00	38.55	54.90
50–59	36.00	37.10	51.45	34.00	39.00	52.45	35.00	39.10	54.45	36.00	37.55	56.00
60–69	30.00	32.00	51.90	29.00	33.65	54.00	26.00	34.00	51.80	26.00	34.55	51.90
70–85	33.00	35.55	53.45	34.00	34.55	53.00	35.00	35.00	53.00	34.00	35.00	54.45
	1.5			1.6			1.7			1.8		
	p1	p5	p95	p1	p5	p95	p1	p5	p95	p1	p5	p95
18–29	38.00	38.55	59.00	35.00	37.10	56.00	33.00	35.65	53.00	33.00	36.55	55.00
30–39	38.00	39.00	59.00	37.00	37.55	56.35	35.00	37.55	53.25	35.00	39.10	55.00
40–49	25.00	36.55	55.35	23.00	34.55	54.25	25.00	34.55	53.00	33.00	36.55	53.80
50–59	36.00	37.00	56.00	33.00	35.55	53.00	34.00	35.00	50.45	35.00	36.55	51.45
60–69	26.00	33.55	50.90	27.00	31.65	52.45	28.00	32.55	49.45	31.00	31.55	48.45
70–85	34.00	35.00	54.00	33.00	34.55	51.90	29.00	34.00	49.00	31.00	33.00	49.00
	2.1			2.2			2.3			2.4		
	p1	p5	p95	p1	p5	p95	p1	p5	p95	p1	p5	p95
18–29	37.00	40.55	58.35	40.00	41.00	59.00	40.00	42.00	62.45	35.00	42.10	63.00
30–39	40.00	41.55	58.45	41.00	41.55	60.90	40.00	43.10	62.90	40.00	42.00	64.45
40–49	25.00	40.00	56.00	29.00	41.55	57.45	32.00	42.10	60.00	35.00	40.65	61.45
50–59	37.00	38.55	53.45	37.00	40.55	56.90	40.00	42.10	60.80	40.00	43.55	64.35
60–69	33.00	34.00	50.45	31.00	36.10	51.45	32.00	38.00	55.00	33.00	38.55	56.45
70–85	34.00	36.55	53.00	36.00	37.55	55.90	36.00	36.55	60.00	34.00	35.55	62.45
	2.5			2.6			2.7			2.8		

	p1	p5	p95	p1	p5	p95	p1	p5	p95	p1	p5	p95
18–29	35.00	41.20	64.45	39.00	39.55	62.90	35.00	37.00	59.00	31.00	34.00	53.00
30–39	40.00	41.00	65.00	39.00	42.55	63.45	38.00	38.55	58.45	34.00	36.10	51.80
40–49	34.00	39.55	61.00	31.00	39.00	57.45	28.00	36.00	55.00	28.00	33.10	52.00
50–59	41.00	42.55	65.35	39.00	40.55	60.00	34.00	35.65	55.45	34.00	35.00	50.45
60–69	34.00	38.10	57.00	33.00	37.65	54.45	30.00	32.65	51.45	29.00	32.00	47.35
70–85	33.00	34.65	63.45	32.00	33.55	63.25	30.00	32.20	54.80	29.00	30.10	48.45
	3.1			3.2			3.3			3.4		
	p1	p5	p95	p1	p5	p95	p1	p5	p95	p1	p5	p95
18–29	40.00	42.10	60.00	44.00	46.10	65.45	48.00	50.10	70.45	35.00	44.10	77.00
30–39	42.00	43.00	62.90	43.00	44.55	64.80	44.00	47.00	70.45	42.00	46.10	75.45
40–49	31.00	39.65	57.45	35.00	42.55	63.00	41.00	44.10	70.45	44.00	47.00	75.25
50–59	38.00	40.00	60.35	42.00	42.55	65.45	41.00	47.20	74.45	41.00	45.20	76.45
60–69	35.00	36.10	53.45	35.00	39.00	57.45	36.00	40.75	64.45	34.00	43.00	72.00
70–85	35.00	37.55	55.90	39.00	39.00	61.35	37.00	39.65	70.00	35.00	36.55	76.80
	3.5			3.6			3.7			3.8		
	p1	p5	p95	p1	p5	p95	p1	p5	p95	p1	p5	p95
18–29	30.00	40.10	76.00	32.00	40.00	72.45	35.00	38.65	68.00	31.00	36.20	59.90
30–39	37.00	40.55	76.45	40.00	41.55	71.90	39.00	42.55	64.90	37.00	37.55	56.45
40–49	40.00	41.65	72.45	39.00	40.55	67.00	32.00	38.10	58.00	26.00	32.65	55.45
50–59	43.00	45.55	73.90	40.00	43.00	68.90	38.00	39.55	62.45	31.00	34.55	55.35
60–69	35.00	40.55	72.45	38.00	39.00	66.35	36.00	37.55	58.45	27.00	30.10	50.45
70–85	31.00	33.55	78.90	32.00	34.00	71.35	29.00	33.55	60.45	28.00	30.55	49.90
	4.1			4.2			4.3			4.4		
	p1	p5	p95	p1	p5	p95	p1	p5	p95	p1	p5	p95
18–29	42.00	44.00	63.45	46.00	49.00	71.00	51.00	52.55	80.90	53.00	55.55	97.00
30–39	41.00	43.00	64.90	42.00	46.00	69.35	46.00	47.65	78.90	55.00	56.10	93.15
40–49	33.00	42.00	62.00	38.00	45.55	69.00	47.00	52.10	80.45	51.00	59.10	95.45
50–59	40.00	42.10	60.35	45.00	46.00	71.35	40.00	44.85	84.00	44.00	52.85	98.35
60–69	35.00	36.00	56.45	37.00	40.00	64.00	38.00	39.55	77.35	45.00	51.10	97.35
70–85	35.00	39.55	57.90	39.00	42.00	67.00	42.00	42.55	80.45	45.00	47.10	97.70
	4.5			4.6			4.7			4.8		
	p1	p5	p95	p1	p5	p95	p1	p5	p95	p1	p5	p95
18–29	41.00	44.10	103.00	31.00	41.20	88.45	32.00	41.10	76.35	31.00	38.00	66.45
30–39	44.00	51.65	95.45	37.00	39.65	82.35	39.00	40.55	70.00	37.00	37.00	57.00
40–49	46.00	52.55	93.00	35.00	41.55	80.45	35.00	38.00	65.00	30.00	31.65	54.45
50–59	55.00	55.55	96.45	38.00	44.10	82.00	37.00	41.00	68.00	29.00	34.10	55.45
60–69	43.00	51.65	96.80	37.00	38.55	81.15	36.00	37.10	66.70	29.00	33.55	53.45
70–85	46.00	49.75	101.45	35.00	37.10	85.90	31.00	36.00	67.80	25.00	26.00	53.45
**ONL**	5.1			5.2			5.3			5.4		
	p1	p5	p95	p1	p5	p95	p1	p5	p95	p1	p5	p95
18–29	40.00	44.55	64.90	43.00	49.55	71.45	50.00	56.10	82.00	55.00	59.65	98.45
30–39	39.00	43.00	68.45	42.00	43.55	70.00	45.00	50.55	80.45	61.00	61.00	92.45
40–49	34.00	42.55	63.45	41.00	46.65	70.35	52.00	55.55	83.35	44.00	60.65	97.90
50–59	37.00	42.10	60.00	43.00	48.00	73.90	45.00	48.65	87.45	55.00	63.55	102.70
60–69	37.00	38.55	57.45	44.00	44.55	67.45	45.00	50.00	83.45	58.00	62.40	100.90
70–85	35.00	40.55	58.80	40.00	44.55	72.00	44.00	52.20	82.45	57.00	64.20	94.35
	5.5			5.6			5.7			5.8		
	p1	p5	p95	p1	p5	p95	p1	p5	p95	p1	p5	p95
18–29	51.00	57.55	106.45	41.00	48.85	88.45	40.00	44.85	77.45	34.00	39.30	66.90
30–39	48.00	50.65	96.00	37.00	38.00	81.00	39.00	41.20	70.45	37.00	38.00	57.00
40–49	50.00	55.00	97.00	41.00	45.55	80.45	40.00	41.55	69.35	32.00	34.00	59.35
50–59	47.00	53.10	99.00	38.00	45.00	84.00	41.00	42.55	71.00	33.00	35.00	57.00
60–69	49.00	55.30	97.45	38.00	41.20	81.35	38.00	41.20	68.35	34.00	34.55	52.45
70–85	48.00	49.55	99.45	37.00	37.00	84.90	37.00	39.00	69.35	32.00	32.55	57.45
	6.1			6.2			6.3			6.4		
	p1	p5	p95	p1	p5	p95	p1	p5	p95	p1	p5	p95
18–29	40.00	45.65	64.00	44.00	49.10	69.45	53.00	54.55	76.00	54.00	56.55	84.90
30–39	42.00	44.00	62.90	42.00	46.10	68.90	40.00	51.55	74.45	38.00	56.65	80.00
40–49	32.00	43.55	61.00	38.00	44.20	67.45	48.00	51.20	74.00	53.00	55.65	80.00
50–59	38.00	42.55	60.45	40.00	46.00	69.80	45.00	53.65	78.25	50.00	57.00	84.00
60–69	38.00	39.55	57.90	43.00	44.00	63.45	45.00	51.55	72.25	44.00	54.55	79.45
70–85	36.00	40.55	57.80	39.00	43.55	65.45	45.00	48.00	72.90	47.00	53.20	79.90

		6.5			6.6			6.7			6.8	
	p1	p5	p95	p1	p5	p95	p1	p5	p95	p1	p5	p95
18–29	48.00	58.00	86.80	46.00	52.65	82.35	44.00	48.00	76.45	41.00	44.55	71.00
30–39	40.00	53.75	80.45	44.00	51.00	77.00	42.00	48.00	71.45	38.00	44.00	64.45
40–49	51.00	57.55	80.00	52.00	52.00	73.90	40.00	46.00	69.00	32.00	40.55	66.45
50–59	45.00	54.40	84.35	45.00	50.10	75.90	44.00	44.55	69.35	37.00	40.20	63.45
60–69	50.00	51.65	79.45	43.00	49.30	73.90	41.00	45.10	66.90	36.00	39.10	60.00
70–85	41.00	43.20	78.80	43.00	46.00	72.35	41.00	42.00	65.00	36.00	37.55	58.45
		7.1			7.2			7.3			7.4	
	p1	p5	p95	p1	p5	p95	p1	p5	p95	p1	p5	p95
18–29	43.00	46.10	63.00	47.00	49.55	66.00	50.00	51.55	70.90	49.00	53.20	76.00
30–39	44.00	44.00	60.90	43.00	46.55	63.45	42.00	48.55	68.00	42.00	52.10	73.45
40–49	30.00	42.20	60.90	33.00	44.55	64.45	38.00	47.65	69.00	44.00	51.10	73.45
50–59	38.00	42.55	59.00	40.00	44.55	64.35	45.00	49.55	71.70	47.00	53.00	75.80
60–69	38.00	39.00	54.80	41.00	41.55	58.90	44.00	44.55	64.80	47.00	50.55	71.90
70–85	35.00	41.00	55.45	37.00	43.10	57.00	41.00	45.00	62.35	45.00	47.00	68.80
		7.5			7.6			7.7			7.8	
	p1	p5	p95	p1	p5	p95	p1	p5	p95	p1	p5	p95
18–29	48.00	52.75	80.25	46.00	51.55	76.35	45.00	49.10	72.45	43.00	45.00	71.35
30–39	42.00	51.55	75.00	43.00	49.00	71.35	42.00	47.55	68.90	38.00	44.20	62.80
40–49	47.00	50.10	73.45	40.00	47.00	70.00	35.00	43.10	67.45	31.00	39.55	66.45
50–59	45.00	51.00	74.00	43.00	47.65	72.25	41.00	45.55	67.80	36.00	38.00	64.00
60–69	48.00	50.55	72.90	47.00	47.55	69.00	41.00	44.10	63.00	37.00	40.00	60.35
70–85	46.00	47.10	70.80	44.00	45.00	65.90	41.00	41.00	63.45	36.00	37.55	57.00
		8.1			8.2			8.3			8.4	
	p1	p5	p95	p1	p5	p95	p1	p5	p95	p1	p5	p95
18–29	44.00	45.65	61.45	45.00	47.75	63.90	45.00	48.75	66.90	46.00	50.20	71.00
30–39	42.00	43.55	59.80	43.00	46.00	61.80	42.00	47.00	64.45	42.00	48.00	68.00
40–49	27.00	41.20	60.45	30.00	41.10	62.90	32.00	44.10	64.45	35.00	45.75	66.45
50–59	37.00	42.55	59.90	40.00	43.10	63.35	41.00	45.65	67.80	41.00	45.65	70.25
60–69	37.00	38.55	56.45	38.00	39.55	59.00	41.00	43.00	62.45	43.00	44.10	64.80
70–85	35.00	39.55	54.45	38.00	41.00	55.00	39.00	43.10	57.00	43.00	43.55	60.00
		8.5			8.6			8.7			8.8	
	p1	p5	p95	p1	p5	p95	p1	p5	p95	p1	p5	p95
18–29	46.00	50.30	72.45	47.00	48.00	71.45	46.00	46.55	70.00	41.00	44.00	65.45
30–39	43.00	47.10	66.90	42.00	46.55	66.35	42.00	46.65	64.45	44.00	44.55	59.45
40–49	36.00	45.20	66.90	33.00	44.20	67.90	29.00	41.65	68.70	27.00	41.55	66.45
50–59	41.00	45.65	68.15	38.00	43.55	66.25	39.00	40.00	64.00	38.00	42.20	61.90
60–69	45.00	46.00	65.25	44.00	45.55	64.45	38.00	40.55	60.00	38.00	41.20	59.45
70–85	42.00	43.00	61.45	41.00	41.55	59.45	37.00	39.10	57.45	38.00	38.00	56.45

Table S7. 1st, 5th and 95th percentiles values of the retinal pigment epithelium (RPE) thickness in each of the 64 cells of the 8 × 8 macular grid in the different age groups.

RPE		**1.1**			**1.2**			**1.3**			**1.4**	
	**p1**	**p5**	**p95**	**p1**	**p5**	**p95**	**p1**	**p5**	**p95**	**p1**	**p5**	**p95**
18–29	9.00	9.55	14.00	9.00	9.55	15.00	9.00	9.55	15.00	9.00	10.00	15.00
30–39	9.00	10.00	14.00	9.00	9.00	15,00	10,00	10,00	15,45	10,00	10,00	15,90
40–49	9.00	9.00	13.45	10.00	10.00	13,45	9,00	10,00	14,00	10,00	10,55	14,00
50–59	10.00	10.00	14.00	10.00	10.00	13,45	10,00	10,00	15,00	9,00	10,00	15,00
60–69	9.00	10.00	12.45	10.00	10.00	13,00	9,00	9,55	13,00	9,00	9,55	14,00
70–85	10.00	10.00	13.45	9.00	10.00	14,00	9,00	9,55	14,45	10,00	10,00	14,45
		1.5			1.6			1.7			1.8	
	p1	p5	p95	p1	p5	p95	p1	p5	p95	p1	p5	p95
18–29	9.00	10.00	15.00	9.00	10.00	15,45	9,00	9,00	14,45	9,00	9,00	18,90
30–39	10.00	10.00	15.00	9.00	10.00	15,45	9,00	9,55	15,00	9,00	9,00	18,90
40–49	10.00	10.00	14.00	9.00	9.55	14,00	9,00	9,00	14,00	8,00	9,00	15,35
50–59	9.00	10.00	14.45	9.00	10.00	14,45	9,00	10,00	14,45	9,00	9,00	14,45
60–69	9.00	9.55	14.00	9.00	9.00	14,00	9,00	9,00	13,00	9,00	9,00	13,45
70–85	9.00	9.00	14.00	9.00	9.00	14,00	9,00	10,00	14,00	9,00	9,55	13,00
		2.1			2.2			2.3			2.4	
	p1	p5	p95	p1	p5	p95	p1	p5	p95	p1	p5	p95
18–29	9.00	9.55	14.00	10.00	10.00	15,00	10,00	10,00	15,45	10,00	10,00	16,00

30–39	10.00	10.00	14.00	9.00	10.00	15,45	9,00	10,00	15,45	10,00	10,55	15,45
40–49	10.00	10.00	13.00	9.00	10.00	13,00	10,00	10,00	14,45	11,00	11,00	15,00
50–59	9.00	9.55	14.00	10.00	10.00	14,45	10,00	11,00	15,00	11,00	11,00	15,00
60–69	9.00	10.00	13.00	9.00	10.00	13,00	10,00	10,00	14,00	9,00	10,00	14,00
70–85	10.00	10.00	14.45	10.00	10.00	14,45	10,00	10,55	15,00	10,00	10,00	15,00
		2.5			2.6			2.7			2.8	
	p1	p5	p95	p1	p5	p95	p1	p5	p95	p1	p5	p95
18–29	10.00	10.55	16.00	10.00	10.00	16,00	9,00	9,55	15,45	9,00	9,00	21,35
30–39	10.00	10.00	16.45	10.00	10.00	17,00	9,00	10,00	16,00	9,00	9,00	18,35
40–49	11.00	11.00	14.90	9.00	10.00	15,45	9,00	10,00	14,00	10,00	10,00	14,45
50–59	10.00	10.55	16.00	9.00	10.00	15,45	9,00	9,00	15,00	9,00	9,55	15,00
60–69	9.00	10.00	15.00	9.00	9.55	15,45	9,00	9,00	14,00	9,00	9,55	14,00
70–85	9.00	10.00	15.00	10.00	10.00	15,45	9,00	10,00	14,45	9,00	9,55	13,45
		3.1			3.2			3.3			3.4	
	p1	p5	p95	p1	p5	p95	p1	p5	p95	p1	p5	p95
18–29	9.00	9.00	14.00	8.00	10.00	14,45	9,00	10,00	15,00	10,00	10,55	16,45
30–39	9.00	10.00	14.00	9.00	10.00	14,45	9,00	10,00	15,45	11,00	11,55	17,00
40–49	10.00	10.55	13.00	10.00	10.55	14,00	11,00	11,00	14,45	11,00	11,00	15,45
50–59	10.00	10.00	14.00	10.00	11.00	15,45	11,00	11,55	15,00	12,00	12,00	16,00
60–69	9.00	10.00	14.00	10.00	10.55	14,00	10,00	10,00	15,45	11,00	11,55	15,45
70–85	10.00	11.00	14.90	10.00	11.00	15,00	11,00	11,00	15,00	11,00	11,00	16,00
		3.5			3.6			3.7			3.8	
	p1	p5	p95	p1	p5	p95	p1	p5	p95	p1	p5	p95
18–29	11.00	11.00	17.00	10.00	11.00	17,00	10,00	10,55	17,00	9,00	9,55	16,45
30–39	11.00	11.55	17.00	10.00	11.00	16,45	10,00	11,00	15,90	10,00	10,00	15,00
40–49	11.00	11.55	15.00	11.00	11.00	15,00	10,00	10,00	14,00	9,00	9,00	14,45
50–59	10.00	11.55	16.45	10.00	10.55	16,00	9,00	10,55	16,00	10,00	10,00	17,80
60–69	11.00	11.00	16.45	10.00	11.00	16,45	10,00	10,00	16,90	9,00	9,00	15,45
70–85	11.00	11.00	16.00	10.00	10.55	16,45	9,00	9,55	15,45	9,00	9,00	14,45
		4.1			4.2			4.3			4.4	
	p1	p5	p95	p1	p5	p95	p1	p5	p95	p1	p5	p95
18–29	9.00	9.00	13.00	9.00	10.00	14,00	10,00	10,55	15,00	12,00	13,00	18,00
30–39	9.00	10.00	14.45	9.00	10.55	15,00	11,00	11,00	16,00	13,00	13,55	18,00
40–49	9.00	10.00	13.45	10.00	11.00	14,00	11,00	12,00	15,45	12,00	13,55	18,00
50–59	10.00	10.55	14.00	11.00	11.00	15,00	11,00	11,55	16,45	13,00	13,00	18,00
60–69	10.00	10.00	14.00	10.00	10.55	14,45	11,00	12,00	17,00	12,00	13,00	18,00
70–85	9.00	11.00	14.45	11.00	11.00	14,00	10,00	11,00	16,00	12,00	12,00	17,45
		4.5			4.6			4.7			4.8	
	p1	p5	p95	p1	p5	p95	p1	p5	p95	p1	p5	p95
18–29	13.00	14.00	19.00	11.00	11.55	17,90	10,00	10,55	16,00	9,00	9,55	18,45
30–39	14.00	14.00	19.45	11.00	11.55	18,90	10,00	11,00	16,90	10,00	10,00	16,45
40–49	13.00	14.00	18.00	11.00	11.55	16,45	10,00	10,00	15,00	9,00	9,00	14,00
50–59	13.00	13.00	19.45	11.00	12.00	18,00	10,00	10,55	16,45	9,00	9,55	16,00
60–69	13.00	13.00	18.45	11.00	12.00	19,00	10,00	11,00	16,45	9,00	9,55	15,00
70–85	12.00	12.55	19.90	11.00	11.00	18,45	10,00	10,00	16,00	9,00	9,00	15,45
**RPE**		5.1			5.2			5.3			5.4	
	p1	p5	p95	p1	p5	p95	p1	p5	p95	p1	p5	p95
18–29	9.00	10.00	13.00	9.00	10.00	14,00	10,00	11,00	15,45	12,00	13,00	18,00
30–39	10.00	10.00	15.90	10.00	10.00	16,00	10,00	11,55	20,70	14,00	14,00	21,70
40–49	9.00	10.00	14.00	10.00	11.00	14,00	12,00	12,00	15,00	13,00	13,00	18,00
50–59	10.00	11.00	14.00	11.00	11.00	15,45	12,00	12,00	16,00	12,00	14,00	18,00
60–69	10.00	10.00	14.45	10.00	11.00	16,00	11,00	12,00	15,90	13,00	13,00	19,00
70–85	10.00	11.00	14.00	11.00	11.00	15,00	11,00	11,00	17,00	11,00	11,55	18,00
		5.5			5.6			5.7			5.8	
	p1	p5	p95	p1	p5	p95	p1	p5	p95	p1	p5	p95
18–29	13.00	13.55	19.45	11.00	12.00	18,00	11,00	11,00	17,00	10,00	10,00	16,00
30–39	13.00	14.00	19.90	10.00	12.00	17,45	10,00	10,55	17,45	9,00	9,00	15,45
40–49	13.00	14.00	18.00	11.00	11.55	16,00	10,00	10,00	15,00	9,00	9,55	15,45
50–59	13.00	13.55	19.00	12.00	12.00	17,00	11,00	11,00	16,00	9,00	10,00	14,90
60–69	13.00	13.55	19.00	12.00	12.00	18,90	10,00	10,00	16,45	10,00	10,00	14,45
70–85	12.00	12.00	18.45	11.00	11.00	17,00	9,00	10,00	16,00	9,00	9,55	14,00
		6.1			6.2			6.3			6.4	
	p1	p5	p95	p1	p5	p95	p1	p5	p95	p1	p5	p95

18–29	9.00	9.00	13.00	9.00	9.55	14,00	10,00	10,00	15,00	10,00	11,00	17,00
30–39	8.00	9.55	14.45	10.00	10.00	15,90	9,00	11,00	16,90	11,00	11,00	17,90
40–49	9.00	10.00	13.00	10.00	11.00	14,00	10,00	11,55	15,00	11,00	12,00	16,00
50–59	10.00	10.00	14.00	11.00	11.00	14,45	11,00	11,00	15,45	11,00	12,00	16,45
60–69	10.00	10.00	13.00	10.00	10.00	15,00	11,00	11,00	15,45	11,00	12,00	17,00
70–85	10.00	10.00	14.45	10.00	11.00	14,45	10,00	10,55	16,45	10,00	11,00	17,45
		6.5			6.6			6.7			6.8	
	p1	p5	p95	p1	p5	p95	p1	p5	p95	p1	p5	p95
18–29	11.00	12.00	18.00	11.00	11.00	17,00	9,00	11,00	17,45	10,00	10,00	16,00
30–39	12.00	12.00	17.45	10.00	11.00	17,00	10,00	11,00	18,00	10,00	10,00	16,00
40–49	12.00	12.55	17.00	11.00	12.00	16,00	10,00	10,55	15,45	9,00	10,00	16,00
50–59	11.00	12.00	18.00	11.00	11.55	17,00	10,00	10,55	16,00	9,00	10,00	17,45
60–69	12.00	12.55	18.00	11.00	11.55	17,45	10,00	10,00	16,00	9,00	10,00	15,00
70–85	11.00	11.55	18.45	10.00	10.00	17,00	9,00	9,00	16,45	9,00	9,00	16,00
		7.1			7.2			7.3			7.4	
	p1	p5	p95	p1	p5	p95	p1	p5	p95	p1	p5	p95
18–29	8.00	9.00	14.00	9.00	9.55	14,00	10,00	10,00	15,45	10,00	10,55	16,00
30–39	9.00	10.00	15.45	9.00	10.00	14,90	10,00	10,55	15,90	10,00	11,00	16,90
40–49	9.00	9.55	13.00	9.00	10.00	13,45	10,00	10,55	14,00	11,00	11,00	15,00
50–59	10.00	10.00	14.45	10.00	10.00	14,45	10,00	10,55	15,00	11,00	11,00	15,00
60–69	9.00	9.55	13.45	10.00	10.00	14,00	10,00	10,55	15,00	11,00	11,00	15,45
70–85	10.00	10.00	14.45	10.00	10.00	14,45	10,00	10,55	16,00	10,00	11,00	15,90
		7.5			7.6			7.7			7.8	
	p1	p5	p95	p1	p5	p95	p1	p5	p95	p1	p5	p95
18–29	9.00	10.00	17.00	9.00	11.00	17,00	8,00	10,00	16,45	9,00	10,00	24,00
30–39	11.00	11.00	17.35	10.00	10.55	17,90	9,00	10,55	18,45	10,00	10,55	17,35
40–49	11.00	12.00	15.45	11.00	11.00	16,00	10,00	10,00	16,00	10,00	10,00	15,00
50–59	10.00	11.55	16.00	9.00	11.00	16,00	9,00	10,55	16,00	10,00	10,00	17,45
60–69	11.00	12.00	16.00	10.00	11.00	16,45	10,00	10,00	15,00	9,00	10,00	14,45
70–85	10.00	11.00	16.90	10.00	10.00	16,00	8,00	10,00	16,00	10,00	10,00	15,00
		8.1			8.2			8.3			8.4	
	p1	p5	p95	p1	p5	p95	p1	p5	p95	p1	p5	p95
18–29	8.00	9.00	14.00	9.00	9.00	14,45	9,00	10,00	15,00	9,00	10,00	15,45
30–39	9.00	10.00	14.45	10.00	10.00	14,45	10,00	10,00	15,80	10,00	10,55	16,80
40–49	10.00	10.00	14.90	9.00	9.55	14,00	9,00	10,00	14,00	10,00	10,55	14,00
50–59	10.00	10.00	14.00	10.00	10.00	14,45	9,00	10,00	15,00	10,00	11,00	15,00
60–69	9.00	9.55	12.45	9.00	10.00	13,45	9,00	9,55	13,45	10,00	10,55	15,00
70–85	10.00	10.00	17.60	10.00	10.00	15,00	9,00	10,00	15,00	10,00	10,00	15,00
		8.5			8.6			8.7			8.8	
	p1	p5	p95	p1	p5	p95	p1	p5	p95	p1	p5	p95
18–29	9.00	10.00	16.00	9.00	10.55	16,00	9,00	9,55	16,45	9,00	9,55	16,90
30–39	10.00	11.00	16.35	10.00	11.00	16,45	9,00	10,00	16,90	10,00	10,55	16,00
40–49	10.00	10.55	15.00	10.00	10.00	15,00	10,00	10,00	15,00	9,00	10,00	14,45
50–59	10.00	11.00	15.45	10.00	10.55	15,00	10,00	10,00	15,00	10,00	10,00	15,00
60–69	10.00	10.00	15.00	10.00	10.00	15,00	10,00	10,00	15,00	9,00	10,00	14,00
70–85	9.00	10.00	16.00	9.00	9.00	16,45	9,00	9,55	15,45	9,00	9,55	15,00

Table S8. 1st, 5th and 95th percentiles values of the INNER retina thickness in each of the 64 cells of the 8 × 8 macular grid in the different age groups.

INNER		**1.1**			**1.2**			**1.3**			**1.4**	
	**p1**	**p5**	**p95**	**p1**	**p5**	**p95**	**p1**	**p5**	**p95**	**p1**	**p5**	**p95**
18–29	143.00	149.55	191.00	153.00	156.20	200.00	160.00	169.10	206.90	166.00	181.20	220.45
30–39	140.00	142.55	178.45	149.00	150.55	191.00	157.00	161.55	200.00	169.00	174.55	217.80
40–49	142.00	143.55	183.00	148.00	153.10	200.35	158.00	164.65	207.80	169.00	174.20	216.70
50–59	138.00	139.00	179.60	147.00	147.00	193.80	153.00	157.85	204.05	163.00	167.85	222.15
60–69	131.00	134.85	170.90	145.00	146.00	185.45	152.00	159.00	200.90	165.00	165.55	215.40
70–85	136.00	139.00	178.35	146.00	147.10	187.80	150.00	155.75	198.80	161.00	164.30	217.90
		1.5			1.6			1.7			1.8	
	p1	p5	p95	p1	p5	p95	p1	p5	p95	p1	p5	p95
18–29	173.00	189.55	235.15	191.00	200.85	254.70	210.00	215.10	265.80	187.00	191.55	251.90
30–39	172.00	177.95	235.00	176.00	186.85	257.00	196.00	198.20	255.15	170.00	181.05	247.00
40–49	179.00	189.65	232.00	186.00	199.00	257.45	204.00	208.55	262.90	183.00	194.10	268.95

50–59	168.00	184.40	241.50	179.00	196.40	261.00	189.00	197.55	258.75	175.00	184.05	252.25
60–69	168.00	173.65	232.15	179.00	181.10	249.35	186.00	193.10	246.00	165.00	174.10	240.50
70–85	170.00	172.10	233.25	180.00	184.10	239.45	190.00	193.10	255.90	176.00	183.85	255.70
	2.1			2.2			2.3			2.4		
	p1	p5	p95	p1	p5	p95	p1	p5	p95	p1	p5	p95
18–29	148.00	153.20	187.45	156.00	165.65	197.00	176.00	179.10	217.90	186.00	188.65	235.70
30–39	144.00	145.00	183.10	155.00	155.55	197.05	167.00	172.65	220.35	185.00	185.00	237.70
40–49	145.00	148.55	182.45	158.00	162.55	195.00	176.00	182.00	220.00	192.00	196.75	233.90
50–59	141.00	142.55	182.35	151.00	157.55	192.45	172.00	179.10	213.45	190.00	192.75	232.00
60–69	143.00	144.10	174.90	154.00	156.55	190.00	165.00	172.10	203.45	179.00	185.55	220.35
70–85	134.00	141.65	179.80	145.00	148.65	196.90	163.00	165.75	219.15	171.00	177.55	243.85
	2.5			2.6			2.7			2.8		
	p1	p5	p95	p1	p5	p95	p1	p5	p95	p1	p5	p95
18–29	191.00	193.20	239.90	189.00	198.95	252.00	197.00	214.10	271.95	221.00	229.10	296.35
30–39	183.00	196.30	246.30	182.00	194.55	255.25	190.00	195.65	277.25	210.00	214.95	282.90
40–49	199.00	202.65	238.00	196.00	201.75	242.45	204.00	209.55	269.25	213.00	228.65	286.95
50–59	195.00	199.85	242.00	197.00	201.00	239.90	197.00	211.95	267.90	211.00	224.85	290.20
60–69	182.00	193.10	230.25	186.00	192.10	239.25	195.00	195.55	255.35	203.00	210.95	265.45
70–85	179.00	183.75	251.80	183.00	183.55	263.30	187.00	192.85	263.95	205.00	207.65	278.80
	3.1			3.2			3.3			3.4		
	p1	p5	p95	p1	p5	p95	p1	p5	p95	p1	p5	p95
18–29	152.00	159.10	192.70	171.00	176.65	218.45	199.00	209.20	257.00	227.00	233.20	284.00
30–39	145.00	156.10	195.60	172.00	177.75	219.45	206.00	209.55	256.05	223.00	236.10	283.45
40–49	157.00	160.65	196.80	176.00	180.55	221.90	208.00	210.55	256.05	234.00	238.75	278.45
50–59	147.00	155.00	189.45	172.00	178.50	217.00	215.00	217.00	253.00	242.00	243.10	278.45
60–69	152.00	155.00	185.90	171.00	173.55	210.90	204.00	207.55	244.15	231.00	233.65	269.95
70–85	143.00	149.30	189.25	159.00	169.55	218.25	183.00	192.85	253.25	209.00	217.55	275.90
	3.5			3.6			3.7			3.8		
	p1	p5	p95	p1	p5	p95	p1	p5	p95	p1	p5	p95
18–29	230.00	239.30	288.00	216.00	226.95	272.80	202.00	212.95	263.30	225.00	227.55	296.60
30–39	217.00	243.55	295.15	213.00	225.10	288.35	198.00	202.30	276.05	194.00	207.55	313.60
40–49	236.00	243.75	283.45	225.00	229.20	270.45	210.00	216.95	255.90	225.00	228.20	281.80
50–59	241.00	244.55	285.90	226.00	231.55	272.80	216.00	217.55	257.25	217.00	225.00	286.80
60–69	232.00	237.65	273.45	213.00	219.30	257.90	196.00	206.55	243.35	201.00	205.20	267.35
70–85	218.00	221.65	278.80	199.00	204.55	272.45	192.00	196.55	255.20	183.00	203.00	282.90
	4.1			4.2			4.3			4.4		
	p1	p5	p95	p1	p5	p95	p1	p5	p95	p1	p5	p95
18–29	158.00	165.30	205.05	183.00	193.00	239.15	221.00	234.65	273.90	193.00	208.00	261.90
30–39	163.00	166.55	200.90	192.00	193.65	239.90	225.00	227.00	279.90	206.00	208.10	261.30
40–49	162.00	168.85	205.45	186.00	195.10	239.45	228.00	229.10	278.90	181.00	206.60	263.45
50–59	157.00	160.30	196.45	187.00	198.10	235.90	232.00	242.10	274.80	192.00	212.40	263.45
60–69	155.00	162.00	191.45	189.00	191.00	230.45	226.00	231.65	272.45	200.00	204.20	257.35
70–85	149.00	150.00	203.05	171.00	175.30	235.45	207.00	212.50	272.45	191.00	200.10	263.90
	4.5			4.6			4.7			4.8		
	p1	p5	p95	p1	p5	p95	p1	p5	p95	p1	p5	p95
18–29	209.00	210.55	267.90	236.00	249.20	292.70	211.00	217.10	265.90	205.00	208.00	267.90
30–39	205.00	207.55	263.25	239.00	249.75	302.90	214.00	215.65	275.15	188.00	199.10	278.45
40–49	196.00	206.65	269.45	237.00	246.30	294.45	221.00	222.65	266.80	206.00	212.10	269.50
50–59	197.00	206.85	276.90	248.00	251.30	289.45	222.00	223.55	267.35	206.00	208.00	261.15
60–69	204.00	204.00	259.90	241.00	244.10	279.45	207.00	212.10	248.25	190.00	194.75	240.90
70–85	199.00	204.00	264.85	226.00	229.20	285.05	195.00	197.55	253.35	190.00	191.00	244.90
**INNER**	5.1			5.2			5.3			5.4		
	p1	p5	p95	p1	p5	p95	p1	p5	p95	p1	p5	p95
18–29	161.00	164.10	203.00	184.00	190.65	237.45	222.00	230.00	272.00	207.00	207.00	260.00
30–39	160.00	165.55	200.45	186.00	189.55	232.45	218.00	221.55	273.00	190.00	198.40	252.15
40–49	162.00	163.10	201.80	181.00	189.55	234.00	218.00	225.55	273.90	203.00	207.20	263.25
50–59	156.00	160.55	197.45	184.00	193.55	236.00	227.00	235.10	273.90	190.00	209.65	256.70
60–69	157.00	161.00	190.45	186.00	186.00	226.45	219.00	227.10	261.45	190.00	196.85	252.45
70–85	146.00	149.55	196.90	169.00	173.10	236.70	205.00	210.65	268.35	175.00	187.55	264.25
	5.5			5.6			5.7			5.8		
	p1	p5	p95	p1	p5	p95	p1	p5	p95	p1	p5	p95
18–29	215.00	220.55	274.25	238.00	253.55	292.90	216.00	223.30	268.80	200.00	214.55	259.45
30–39	199.00	206.20	264.00	238.00	247.55	301.45	210.00	217.55	275.95	179.00	197.85	270.80

40–49	192.00	216.55	274.80	240.00	250.40	295.90	218.00	223.10	269.80	189.00	206.10	266.05
50–59	201.00	212.20	276.35	245.00	253.00	288.35	220.00	226.10	269.80	203.00	205.10	260.80
60–69	204.00	211.55	260.90	241.00	245.85	281.25	204.00	212.10	247.45	184.00	192.95	236.90
70–85	190.00	201.10	276.35	223.00	227.75	281.90	197.00	200.00	256.60	192.00	192.55	262.10
		6.1			6.2			6.3			6.4	
	p1	p5	p95	p1	p5	p95	p1	p5	p95	p1	p5	p95
18–29	155.00	157.00	193.25	175.00	179.00	219.45	204.00	208.85	254.00	231.00	239.65	282.45
30–39	155.00	159.10	193.35	177.00	179.20	219.95	204.00	209.10	254.70	227.00	239.10	282.15
40–49	155.00	156.65	195.35	172.00	180.85	219.35	200.00	204.65	256.00	232.00	233.65	284.00
50–59	150.00	155.55	193.45	174.00	176.65	224.45	209.00	212.65	255.60	242.00	242.55	285.25
60–69	150.00	154.65	185.35	176.00	178.55	210.45	204.00	209.65	242.90	229.00	231.10	274.35
70–85	145.00	148.20	193.95	162.00	167.10	219.70	186.00	189.20	252.35	202.00	213.30	278.70
		6.5			6.6			6.7			6.8	
	p1	p5	p95	p1	p5	p95	p1	p5	p95	p1	p5	p95
18–29	237.00	243.75	292.70	223.00	229.10	277.00	214.00	218.10	257.90	190.00	217.50	273.25
30–39	230.00	245.10	296.90	220.00	226.55	279.80	198.00	208.75	269.05	194.00	201.95	284.60
40–49	235.00	244.65	293.35	225.00	227.00	277.90	180.00	214.20	257.35	155.00	214.60	277.90
50–59	247.00	248.10	288.35	232.00	236.55	273.15	218.00	221.00	256.80	210.00	215.55	282.80
60–69	239.00	240.55	279.35	225.00	226.55	258.90	203.00	210.55	243.80	200.00	205.65	267.40
70–85	202.00	218.10	282.30	202.00	203.00	267.40	193.00	200.00	251.35	199.00	203.65	267.55
		7.1			7.2			7.3			7.4	
	p1	p5	p95	p1	p5	p95	p1	p5	p95	p1	p5	p95
18–29	148.00	152.00	182.35	163.00	166.20	201.35	181.00	183.75	219.45	196.00	198.65	239.00
30–39	144.00	149.30	183.85	156.00	162.65	201.40	171.00	176.65	223.95	193.00	195.55	246.80
40–49	150.00	151.00	180.45	162.00	164.55	198.00	181.00	182.00	221.90	196.00	199.00	244.25
50–59	137.00	147.10	184.90	154.00	161.00	199.00	176.00	180.00	224.60	198.00	202.10	245.90
60–69	141.00	147.55	174.80	157.00	159.55	189.80	179.00	180.00	209.45	197.00	198.00	231.90
70–85	132.00	141.55	181.85	150.00	155.10	198.65	168.00	171.55	215.30	173.00	182.20	236.05
		7.5			7.6			7.7			7.8	
	p1	p5	p95	p1	p5	p95	p1	p5	p95	p1	p5	p95
18–29	204.00	209.30	252.35	206.00	207.55	247.45	209.00	211.00	249.90	217.00	221.85	276.90
30–39	198.00	204.10	254.45	196.00	198.00	249.80	189.00	194.75	254.60	194.00	197.10	281.25
40–49	204.00	207.20	252.80	204.00	206.10	249.90	198.00	204.95	255.70	208.00	212.20	272.45
50–59	209.00	211.65	250.90	205.00	207.00	247.45	200.00	205.00	253.00	205.00	215.75	288.00
60–69	204.00	206.10	238.45	197.00	199.10	237.00	189.00	197.10	238.60	187.00	205.10	265.60
70–85	173.00	185.65	240.25	164.00	188.85	238.40	183.00	191.20	244.95	198.00	203.30	274.50
		8.1			8.2			8.3			8.4	
	p1	p5	p95	p1	p5	p95	p1	p5	p95	p1	p5	p95
18–29	141.00	146.55	178.25	151.00	154.20	188.45	163.00	164.10	203.70	175.00	179.75	212.45
30–39	139.00	140.00	170.90	149.00	153.10	184.90	157.00	160.00	201.15	167.00	170.75	214.15
40–49	141.00	142.55	176.90	152.00	155.00	189.90	159.00	164.10	201.90	173.00	176.10	214.00
50–59	134.00	138.85	171.45	142.00	153.10	181.80	156.00	162.55	199.50	174.00	174.55	215.80
60–69	134.00	139.55	165.35	146.00	149.10	172.45	158.00	161.10	189.45	168.00	171.00	208.45
70–85	130.00	136.65	178.80	137.00	145.20	185.75	149.00	154.20	199.30	163.00	166.55	213.75
		8.5			8.6			8.7			8.8	
	p1	p5	p95	p1	p5	p95	p1	p5	p95	p1	p5	p95
18–29	186.00	188.55	224.00	190.00	197.65	236.45	196.00	208.85	251.45	205.00	214.60	266.80
30–39	172.00	176.30	227.45	177.00	179.20	239.60	182.00	187.30	253.95	200.00	204.10	260.80
40–49	182.00	183.55	225.90	185.00	187.55	237.80	188.00	194.00	251.70	186.00	198.95	263.70
50–59	183.00	185.00	231.80	183.00	187.30	237.80	186.00	197.85	255.00	203.00	207.55	271.90
60–69	180.00	181.55	214.90	180.00	181.55	224.00	182.00	188.55	243.35	183.00	202.15	264.00
70–85	173.00	174.65	222.30	177.00	181.10	232.95	183.00	193.05	252.30	193.00	198.20	263.00

Table S9. 1st, 5th and 95th percentiles values of the OUTER retina thickness in each of the 64 cells of the 8 × 8 macular grid in the different age groups.

OUTER		1.1			1.2			1.3			1.4	
	p1	p5	p95	p1	p5	p95	p1	p5	p95	p1	p5	p95
18–29	70.00	71.55	80.90	71.00	71.55	80.90	71.00	72.00	81.45	71.00	72.00	81.45
30–39	70.00	71.55	80.00	69.00	72.00	80.00	70.00	71.55	80.45	71.00	72.00	80.00
40–49	68.00	69.55	78.00	68.00	70.10	78.45	68.00	69.55	79.00	69.00	70.10	79.00
50–59	68.00	70.10	79.00	69.00	71.55	79.90	69.00	71.10	80.00	69.00	69.55	80.00
60–69	68.00	69.00	78.00	68.00	69.00	78.00	67.00	69.00	78.00	68.00	69.00	79.00

70–85	69.00	69.00	79.00	69.00	69.00	78.45	69.00	69.00	79.00	69.00	69.55	80.00
		1.5			1.6			1.7			1.8	
	p1	p5	p95	p1	p5	p95	p1	p5	p95	p1	p5	p95
18–29	71.00	72.00	81.90	71.00	72.00	81.45	71.00	71.55	82.45	71.00	71.00	84.80
30–39	72.00	72.00	80.00	71.00	71.55	79.00	71.00	72.00	79.45	70.00	71.00	83.00
40–49	69.00	69.55	79.45	68.00	69.55	78.00	68.00	69.55	79.00	67.00	68.55	81.45
50–59	68.00	70.10	79.45	68.00	70.10	80.45	68.00	69.10	80.00	69.00	69.00	78.45
60–69	67.00	69.00	79.00	68.00	69.55	79.45	68.00	69.00	78.00	67.00	69.00	79.00
70–85	68.00	69.00	80.00	68.00	69.00	79.00	68.00	68.55	78.00	68.00	68.00	78.45
		2.1			2.2			2.3			2.4	
	p1	p5	p95	p1	p5	p95	p1	p5	p95	p1	p5	p95
18–29	70.00	71.55	81.45	71.00	72.00	82.35	71.00	72.55	83.00	72.00	72.55	82.90
30–39	71.00	71.55	80.00	70.00	72.00	81.00	71.00	72.00	81.45	73.00	73.00	82.00
40–49	70.00	72.00	79.00	71.00	72.00	80.00	72.00	72.00	80.45	71.00	73.00	81.00
50–59	70.00	71.00	79.45	71.00	71.55	80.45	72.00	72.00	81.00	71.00	72.00	81.45
60–69	70.00	71.00	78.45	71.00	71.55	78.45	72.00	72.00	79.00	72.00	72.00	79.45
70–85	70.00	70.55	79.45	70.00	70.00	80.00	70.00	70.55	80.45	69.00	71.00	80.00
		2.5			2.6			2.7			2.8	
	p1	p5	p95	p1	p5	p95	p1	p5	p95	p1	p5	p95
18–29	71.00	72.00	82.45	72.00	72.55	82.45	71.00	72.55	82.90	71.00	72.55	85.45
30–39	72.00	72.00	81.90	71.00	72.55	82.00	71.00	72.00	80.00	71.00	71.00	81.45
40–49	71.00	72.55	80.45	70.00	71.55	81.00	71.00	72.00	79.00	71.00	72.00	80.00
50–59	69.00	72.00	81.90	69.00	71.55	82.45	69.00	70.55	81.00	68.00	69.55	80.45
60–69	71.00	71.55	80.00	69.00	71.00	81.00	68.00	71.00	79.45	68.00	71.00	79.45
70–85	70.00	71.00	81.00	69.00	70.55	80.00	69.00	69.55	79.00	68.00	69.00	78.00
		3.1			3.2			3.3			3.4	
	p1	p5	p95	p1	p5	p95	p1	p5	p95	p1	p5	p95
18–29	71.00	71.55	82.00	72.00	72.00	82.00	71.00	73.00	83.45	73.00	74.00	84.45
30–39	71.00	71.55	80.45	71.00	72.00	81.45	72.00	72.55	84.90	74.00	74.00	86.70
40–49	71.00	72.00	80.00	72.00	72.00	80.45	73.00	73.00	82.00	73.00	74.00	83.00
50–59	71.00	72.00	80.90	73.00	73.00	82.00	73.00	74.00	81.00	74.00	74.00	82.00
60–69	71.00	72.00	79.00	72.00	73.00	80.00	72.00	72.55	81.45	74.00	74.00	81.00
70–85	70.00	71.10	81.35	70.00	71.55	80.00	71.00	72.10	81.00	73.00	73.00	82.00
		3.5			3.6			3.7			3.8	
	p1	p5	p95	p1	p5	p95	p1	p5	p95	p1	p5	p95
18–29	73.00	74.00	84.00	74.00	74.00	84.00	72.00	74.00	83.00	72.00	72.55	82.45
30–39	74.00	74.00	87.15	73.00	74.00	83.45	72.00	73.00	82.45	71.00	72.00	81.00
40–49	73.00	74.00	82.45	73.00	73.55	81.45	72.00	73.00	79.45	71.00	71.55	79.00
50–59	74.00	74.00	82.45	72.00	73.00	82.00	70.00	72.55	82.00	71.00	71.00	84.50
60–69	73.00	73.55	82.45	72.00	72.00	82.45	70.00	71.55	82.00	69.00	71.00	80.90
70–85	73.00	73.00	82.45	71.00	73.00	82.45	70.00	72.00	81.00	70.00	71.00	81.00
		4.1			4.2			4.3			4.4	
	p1	p5	p95	p1	p5	p95	p1	p5	p95	p1	p5	p95
18–29	71.00	72.00	82.00	72.00	72.55	83.00	73.00	74.00	84.45	79.00	80.00	90.00
30–39	72.00	72.55	81.00	73.00	74.00	85.70	74.00	75.55	90.30	79.00	80.00	91.00
40–49	71.00	72.55	80.45	71.00	73.55	82.00	73.00	75.00	83.00	79.00	80.00	87.45
50–59	72.00	72.00	81.90	73.00	73.00	83.00	74.00	75.00	83.45	79.00	79.00	87.90
60–69	72.00	72.00	80.00	72.00	73.00	80.00	74.00	74.00	83.00	77.00	77.00	90.45
70–85	70.00	72.55	80.00	71.00	73.00	82.00	72.00	74.00	82.45	74.00	76.55	86.90
		4.5			4.6			4.7			4.8	
	p1	p5	p95	p1	p5	p95	p1	p5	p95	p1	p5	p95
18–29	80.00	80.55	89.45	76.00	77.00	87.45	74.00	75.00	84.45	73.00	74.55	85.45
30–39	79.00	80.00	92.00	75.00	76.00	88.70	72.00	74.55	84.00	72.00	73.00	82.00
40–49	80.00	81.00	89.00	75.00	75.55	83.00	73.00	74.00	81.00	73.00	73.00	80.45
50–59	79.00	79.00	88.00	75.00	75.00	84.45	73.00	73.00	83.45	70.00	72.00	82.00
60–69	77.00	78.10	91.45	74.00	74.00	86.45	72.00	73.00	84.45	71.00	72.55	80.45
70–85	76.00	76.00	89.00	74.00	75.00	85.00	72.00	73.55	82.45	71.00	72.00	81.45
**OUTER**		5.1			5.2			5.3			5.4	
	p1	p5	p95	p1	p5	p95	p1	p5	p95	p1	p5	p95
18–29	71.00	71.55	82.45	71.00	72.00	83.45	73.00	74.55	85.00	79.00	79.55	89.00
30–39	73.00	73.55	82.45	74.00	75.00	88.50	76.00	76.00	98.60	80.00	81.00	98.45
40–49	71.00	74.00	81.00	72.00	74.00	82.45	74.00	74.55	84.00	76.00	78.00	88.45
50–59	72.00	73.00	82.45	73.00	73.55	84.00	75.00	76.00	84.45	79.00	80.00	89.00

60–69	72.00	72.00	80.45	73.00	73.00	82.00	74.00	74.55	83.00	77.00	77.55	88.45
70–85	70.00	72.00	80.90	70.00	72.55	83.00	73.00	74.00	85.00	74.00	75.55	87.90
		5.5			5.6			5.7			5.8	
	p1	p5	p95	p1	p5	p95	p1	p5	p95	p1	p5	p95
18–29	79.00	80.55	90.90	76.00	76.55	86.45	75.00	75.55	86.00	75.00	76.00	83.90
30–39	81.00	81.00	94.95	76.00	77.00	91.85	72.00	75.10	84.45	70.00	73.00	81.45
40–49	77.00	79.55	88.45	74.00	75.55	84.00	73.00	74.00	81.00	72.00	73.00	80.45
50–59	79.00	79.00	90.00	75.00	76.00	85.45	73.00	74.00	84.00	71.00	72.55	82.00
60–69	77.00	78.00	89.00	74.00	74.55	85.90	72.00	73.00	84.45	71.00	72.55	81.90
70–85	75.00	76.00	88.90	74.00	75.00	85.00	72.00	74.00	82.00	71.00	72.00	80.45
		6.1			6.2			6.3			6.4	
	p1	p5	p95	p1	p5	p95	p1	p5	p95	p1	p5	p95
18–29	72.00	72.00	82.00	71.00	72.00	83.45	72.00	74.00	84.00	73.00	75.55	86.00
30–39	71.00	73.00	81.90	72.00	73.55	83.45	72.00	74.10	85.35	74.00	74.55	85.35
40–49	71.00	71.55	82.00	72.00	72.55	82.00	73.00	74.00	83.00	74.00	74.55	84.00
50–59	72.00	72.55	81.00	73.00	74.00	82.00	74.00	75.00	83.00	74.00	75.55	84.00
60–69	72.00	72.00	80.00	72.00	72.55	81.00	73.00	74.00	81.90	74.00	74.55	83.45
70–85	72.00	72.00	81.90	72.00	73.00	82.00	73.00	73.55	83.45	73.00	74.00	85.00
		6.5			6.6			6.7			6.8	
	p1	p5	p95	p1	p5	p95	p1	p5	p95	p1	p5	p95
18–29	74.00	75.00	86.00	73.00	74.10	84.45	73.00	74.55	85.45	73.00	74.00	84.90
30–39	75.00	75.55	85.90	73.00	75.55	83.45	72.00	74.55	84.00	71.00	73.55	82.00
40–49	74.00	75.00	84.00	74.00	74.00	82.90	73.00	73.55	82.45	73.00	73.00	82.00
50–59	75.00	75.55	85.00	74.00	75.55	83.45	72.00	74.00	83.45	72.00	72.55	85.90
60–69	73.00	75.00	85.00	74.00	74.00	84.00	72.00	72.55	83.45	71.00	72.00	82.45
70–85	73.00	74.55	85.45	74.00	74.55	83.45	72.00	72.55	83.00	71.00	72.00	82.00
		7.1			7.2			7.3			7.4	
	p1	p5	p95	p1	p5	p95	p1	p5	p95	p1	p5	p95
18–29	72.00	72.55	83.45	72.00	73.00	84.00	73.00	75.10	85.00	73.00	75.55	84.45
30–39	72.00	73.55	82.00	72.00	74.00	81.90	73.00	74.10	83.00	73.00	74.55	84.00
40–49	70.00	71.55	80.90	71.00	72.55	81.00	72.00	74.00	82.00	73.00	74.00	82.45
50–59	72.00	72.55	83.00	73.00	73.00	83.00	72.00	74.00	83.00	73.00	74.00	84.00
60–69	71.00	71.55	80.45	71.00	72.00	81.00	72.00	73.00	81.45	73.00	73.00	83.00
70–85	70.00	72.00	81.45	71.00	72.00	81.45	71.00	73.00	82.35	72.00	73.00	83.35
		7.5			7.6			7.7			7.8	
	p1	p5	p95	p1	p5	p95	p1	p5	p95	p1	p5	p95
18–29	73.00	75.00	84.90	73.00	74.00	86.00	74.00	74.55	85.45	74.00	75.55	89.80
30–39	74.00	75.00	83.90	73.00	75.55	83.90	73.00	75.00	85.00	73.00	75.10	83.35
40–49	74.00	74.00	83.45	73.00	74.00	82.00	72.00	73.55	82.00	72.00	73.00	81.00
50–59	74.00	74.55	84.00	73.00	74.00	85.00	73.00	73.00	84.00	72.00	72.55	83.90
60–69	73.00	73.55	83.45	73.00	73.55	83.45	71.00	72.00	83.90	70.00	71.55	83.45
70–85	72.00	73.00	84.90	70.00	70.55	83.90	70.00	71.10	82.45	70.00	71.65	81.45
		8.1			8.2			8.3			8.4	
	p1	p5	p95	p1	p5	p95	p1	p5	p95	p1	p5	p95
18–29	72.00	73.55	84.00	72.00	73.55	83.45	72.00	74.55	84.00	72.00	73.55	83.45
30–39	72.00	73.00	80.45	72.00	74.00	80.90	72.00	74.55	83.45	74.00	74.55	83.45
40–49	70.00	72.00	81.45	71.00	71.55	81.45	71.00	72.55	82.00	71.00	72.55	82.45
50–59	71.00	72.00	82.45	72.00	72.55	82.45	72.00	73.00	83.45	73.00	74.00	83.00
60–69	71.00	71.55	80.00	71.00	71.55	80.00	71.00	72.00	80.45	72.00	72.55	82.00
70–85	69.00	70.00	83.35	71.00	71.00	81.00	70.00	70.00	80.45	69.00	69.55	81.45
		8.5			8.6			8.7			8.8	
	p1	p5	p95	p1	p5	p95	p1	p5	p95	p1	p5	p95
18–29	73.00	74.55	85.00	73.00	73.55	84.90	72.00	73.65	85.45	72.00	73.65	85.80
30–39	75.00	76.00	83.45	74.00	76.00	83.00	73.00	75.00	83.35	73.00	74.00	82.00
40–49	72.00	73.00	82.00	72.00	72.55	81.45	71.00	72.55	81.00	71.00	72.55	81.45
50–59	72.00	74.00	82.45	73.00	73.55	83.00	72.00	72.55	83.45	72.00	72.00	82.90
60–69	72.00	72.00	82.00	72.00	72.55	82.00	71.00	72.00	83.00	71.00	72.55	81.90
70–85	69.00	70.65	82.90	68.00	68.55	82.45	68.00	69.65	81.90	68.00	69.65	80.90

Table S10. 1st, 5th and 95th percentiles values of the full RETINA thickness in each of the 64 cells of the 8 × 8 macular grid in the different age groups.

RETINA	1.1	1.2	1.3	1.4

	**p1**	**p5**	**p95**	**p1**	**p5**	**p95**	**p1**	**p5**	**p95**	**p1**	**p5**	**p95**
18–29	213.00	228.00	266.35	229.00	232.20	277.60	236.00	246.10	285.25	242.00	256.65	299.15
30–39	213.00	219.10	252.45	222.00	226.55	266.45	235.00	237.00	277.45	247.00	249.55	293.45
40–49	214.00	216.00	256.35	222.00	225.20	277.45	233.00	235.55	284.70	242.00	245.55	295.25
50–59	211.00	212.10	259.35	219.00	221.10	268.60	226.00	231.65	279.50	237.00	241.20	297.50
60–69	205.00	208.20	245.35	219.00	222.00	260.45	229.00	232.55	276.45	235.00	240.00	292.05
70–85	209.00	211.20	259.70	216.00	219.00	262.35	223.00	230.10	272.45	232.00	238.55	292.35
		1.5			1.6			1.7			1.8	
	p1	p5	p95	p1	p5	p95	p1	p5	p95	p1	p5	p95
18–29	250.00	265.55	315.45	269.00	276.85	330.15	289.00	291.10	343.60	269.00	269.55	328.00
30–39	247.00	254.30	308.80	250.00	264.05	330.35	268.00	271.75	330.45	244.00	256.20	322.35
40–49	252.00	262.10	307.25	259.00	269.75	331.45	276.00	279.20	336.00	256.00	267.10	345.05
50–59	239.00	259.30	319.50	250.00	272.55	336.45	259.00	270.75	335.30	247.00	257.30	327.35
60–69	238.00	246.55	309.80	248.00	251.55	323.45	257.00	263.65	321.90	237.00	244.20	314.95
70–85	240.00	249.20	308.05	255.00	261.55	315.35	265.00	268.55	331.35	244.00	257.60	329.70
		2.1			2.2			2.3			2.4	
	p1	p5	p95	p1	p5	p95	p1	p5	p95	p1	p5	p95
18–29	221.00	227.40	263.45	228.00	240.65	277.00	249.00	256.10	296.80	264.00	264.00	312.90
30–39	217.00	220.55	266.90	228.00	232.00	277.45	241.00	249.10	298.00	260.00	261.55	317.00
40–49	219.00	222.55	259.35	231.00	235.65	273.00	251.00	254.00	296.90	267.00	273.00	310.80
50–59	214.00	216.55	259.45	227.00	231.10	271.45	248.00	253.10	290.80	264.00	268.55	309.45
60–69	217.00	218.55	251.35	230.00	231.55	266.90	237.00	246.00	281.45	252.00	263.10	296.25
70–85	205.00	216.00	254.90	220.00	222.65	269.45	237.00	241.10	294.40	246.00	254.55	318.95
		2.5			2.6			2.7			2.8	
	p1	p5	p95	p1	p5	p95	p1	p5	p95	p1	p5	p95
18–29	265.00	270.75	320.35	268.00	271.85	331.70	276.00	288.00	354.25	297.00	307.55	374.90
30–39	258.00	273.30	323.05	257.00	272.10	331.90	264.00	274.10	351.05	283.00	292.15	359.35
40–49	274.00	280.55	316.00	271.00	277.65	321.80	279.00	285.10	344.45	284.00	302.20	363.60
50–59	269.00	273.55	319.00	269.00	272.00	318.45	269.00	290.55	345.60	282.00	301.00	367.85
60–69	254.00	269.10	304.90	263.00	265.55	315.45	267.00	270.55	331.60	277.00	282.40	338.70
70–85	253.00	260.30	328.45	258.00	259.55	340.65	262.00	268.65	340.85	281.00	284.10	352.80
		3.1			3.2			3.3			3.4	
	p1	p5	p95	p1	p5	p95	p1	p5	p95	p1	p5	p95
18–29	224.00	234.00	269.90	242.00	253.10	296.45	271.00	286.65	335.60	301.00	312.55	362.35
30–39	222.00	231.55	278.25	249.00	254.20	299.35	281.00	287.00	334.45	299.00	316.85	363.00
40–49	231.00	234.20	271.70	247.00	254.20	298.90	282.00	285.10	333.70	313.00	318.20	358.90
50–59	223.00	229.65	269.00	248.00	257.00	296.90	291.00	292.00	334.00	316.00	319.10	359.45
60–69	227.00	230.10	263.80	246.00	248.55	288.25	280.00	282.55	321.15	308.00	308.55	347.70
70–85	216.00	221.75	267.00	234.00	242.75	294.35	259.00	267.05	331.05	287.00	293.65	352.60
		3.5			3.6			3.7			3.8	
	p1	p5	p95	p1	p5	p95	p1	p5	p95	p1	p5	p95
18–29	304.00	319.40	366.45	290.00	304.85	352.00	276.00	290.20	343.40	298.00	305.20	375.05
30–39	293.00	322.55	375.05	290.00	303.75	366.25	272.00	280.40	353.15	269.00	285.55	391.15
40–49	316.00	322.65	363.00	304.00	307.00	349.90	288.00	293.65	334.35	299.00	305.10	357.90
50–59	318.00	319.00	363.45	300.00	306.00	352.15	288.00	295.55	339.45	289.00	303.20	368.45
60–69	308.00	314.55	348.45	286.00	296.75	334.90	269.00	280.65	318.80	272.00	281.10	343.35
70–85	295.00	297.55	356.35	276.00	280.20	352.35	266.00	270.20	334.00	265.00	281.55	362.00
		4.1			4.2			4.3			4.4	
	p1	p5	p95	p1	p5	p95	p1	p5	p95	p1	p5	p95
18–29	230.00	239.75	282.00	255.00	270.10	318.45	294.00	313.85	356.90	276.00	290.55	343.45
30–39	240.00	244.00	284.95	268.00	269.00	319.15	304.00	307.00	360.90	289.00	292.55	346.95
40–49	232.00	243.30	283.45	257.00	272.55	318.35	301.00	308.00	360.25	268.00	287.95	349.90
50–59	230.00	235.65	273.90	261.00	273.10	316.35	308.00	321.10	358.35	279.00	296.85	347.80
60–69	233.00	237.10	269.00	265.00	268.00	307.80	304.00	311.10	350.00	283.00	286.75	339.90
70–85	219.00	223.10	278.80	242.00	250.95	311.25	281.00	290.05	352.35	271.00	280.20	344.35
		4.5			4.6			4.7			4.8	
	p1	p5	p95	p1	p5	p95	p1	p5	p95	p1	p5	p95
18–29	293.00	294.55	357.00	312.00	329.95	376.80	285.00	294.10	347.00	281.00	289.75	344.45
30–39	289.00	294.30	350.80	319.00	331.55	382.80	292.00	295.55	353.05	269.00	278.10	355.00
40–49	283.00	293.10	355.00	321.00	326.85	373.45	296.00	299.10	346.80	279.00	287.65	345.95
50–59	285.00	291.95	363.35	324.00	333.20	370.45	296.00	299.85	348.00	279.00	284.10	337.25
60–69	287.00	288.10	341.00	319.00	322.10	359.80	282.00	287.10	328.35	261.00	269.20	318.45
70–85	287.00	288.00	346.35	307.00	309.20	364.45	274.00	276.00	334.15	268.00	269.00	324.00

RETINA	5.1			5.2			5.3			5.4		
	p1	p5	p95	p1	p5	p95	p1	p5	p95	p1	p5	p95
18–29	233.00	240.20	279.45	256.00	266.00	315.35	295.00	309.65	352.90	287.00	292.10	343.70
30–39	240.00	242.55	280.70	264.00	265.55	314.25	299.00	303.65	353.90	274.00	282.40	343.60
40–49	233.00	239.55	278.80	253.00	267.10	313.90	292.00	305.65	354.25	288.00	290.55	347.80
50–59	229.00	233.55	276.35	258.00	269.10	316.35	303.00	314.30	354.45	278.00	291.10	343.35
60–69	234.00	236.10	268.80	261.00	265.65	305.25	297.00	308.00	342.00	274.00	279.40	335.80
70–85	215.00	223.10	273.80	239.00	248.65	312.35	283.00	286.20	350.45	256.00	269.50	346.35
	5.5			5.6			5.7			5.8		
	p1	p5	p95	p1	p5	p95	p1	p5	p95	p1	p5	p95
18–29	296.00	304.65	361.90	314.00	332.00	374.45	291.00	303.85	350.35	282.00	294.20	342.90
30–39	281.00	291.65	356.45	319.00	328.10	382.45	290.00	295.55	360.35	258.00	276.95	349.70
40–49	276.00	299.65	359.25	320.00	328.75	373.90	296.00	299.85	348.00	263.00	284.20	346.00
50–59	291.00	301.10	363.60	322.00	334.00	371.35	294.00	304.20	350.90	273.00	281.20	338.35
60–69	286.00	294.00	346.80	319.00	322.10	362.35	284.00	289.55	327.90	260.00	270.30	311.45
70–85	279.00	281.65	356.70	299.00	306.85	361.60	274.00	278.65	336.85	268.00	270.00	341.00
	6.1			6.2			6.3			6.4		
	p1	p5	p95	p1	p5	p95	p1	p5	p95	p1	p5	p95
18–29	229.00	233.10	270.90	247.00	256.10	301.45	276.00	286.95	334.00	304.00	318.75	363.35
30–39	233.00	236.55	276.50	253.00	256.10	304.45	283.00	287.20	336.45	307.00	317.55	362.80
40–49	229.00	232.55	275.45	245.00	256.65	297.35	273.00	280.00	335.45	306.00	313.30	365.35
50–59	223.00	229.55	271.90	248.00	254.65	303.90	286.00	290.10	336.35	318.00	322.55	365.35
60–69	228.00	231.55	264.45	253.00	255.55	290.45	282.00	285.30	321.45	307.00	310.55	354.00
70–85	218.00	222.75	272.30	236.00	243.00	299.15	261.00	265.85	331.50	278.00	290.95	359.30
	6.5			6.6			6.7			6.8		
	p1	p5	p95	p1	p5	p95	p1	p5	p95	p1	p5	p95
18–29	311.00	324.55	374.85	297.00	309.10	359.45	293.00	298.20	337.35	269.00	297.65	354.90
30–39	310.00	325.55	377.45	300.00	305.10	361.35	276.00	287.65	348.60	271.00	279.85	364.15
40–49	314.00	324.55	375.60	303.00	305.65	356.45	253.00	294.00	338.35	227.00	290.60	356.45
50–59	324.00	329.20	369.45	310.00	312.55	355.80	295.00	299.55	338.00	289.00	294.00	359.60
60–69	318.00	318.55	361.00	299.00	306.65	340.90	274.00	287.55	324.35	272.00	282.00	340.50
70–85	279.00	297.30	364.00	278.00	281.10	347.65	272.00	276.20	331.15	279.00	281.55	352.30
	7.1			7.2			7.3			7.4		
	p1	p5	p95	p1	p5	p95	p1	p5	p95	p1	p5	p95
18–29	220.00	231.10	259.45	235.00	244.30	279.45	254.00	263.75	303.45	270.00	277.55	319.35
30–39	221.00	226.20	270.45	234.00	238.65	287.00	248.00	255.00	305.15	272.00	273.65	325.25
40–49	224.00	226.00	259.45	234.00	239.65	276.00	253.00	258.65	300.90	276.00	277.00	323.80
50–59	213.00	221.55	263.25	232.00	235.55	278.45	255.00	256.00	304.35	275.00	278.65	324.90
60–69	220.00	224.00	252.25	234.00	238.00	267.35	256.00	258.55	288.45	271.00	275.55	313.45
70–85	204.00	217.55	258.20	222.00	232.55	277.20	241.00	246.20	294.65	251.00	256.00	315.85
	7.5			7.6			7.7			7.8		
	p1	p5	p95	p1	p5	p95	p1	p5	p95	p1	p5	p95
18–29	279.00	286.65	332.60	279.00	288.00	330.35	285.00	287.55	329.80	296.00	298.75	355.90
30–39	279.00	283.55	335.80	277.00	278.00	335.25	269.00	274.75	335.05	273.00	276.55	360.35
40–49	284.00	285.55	334.00	282.00	284.55	328.70	275.00	281.40	332.70	285.00	290.10	351.00
50–59	286.00	290.20	332.45	282.00	285.10	325.45	280.00	282.00	335.00	283.00	291.65	367.45
60–69	280.00	280.55	318.00	274.00	276.20	316.70	266.00	270.20	318.05	264.00	278.75	345.30
70–85	252.00	262.55	322.60	243.00	265.55	321.30	261.00	268.10	325.30	278.00	281.85	354.75
	8.1			8.2			8.3			8.4		
	p1	p5	p95	p1	p5	p95	p1	p5	p95	p1	p5	p95
18–29	213.00	224.10	255.70	223.00	234.10	266.45	235.00	243.85	281.80	247.00	256.75	293.35
30–39	213.00	217.65	257.45	225.00	229.65	271.40	234.00	237.00	285.25	245.00	248.20	296.45
40–49	211.00	216.20	256.00	228.00	229.55	266.45	237.00	240.20	279.90	252.00	252.00	294.45
50–59	211.00	217.65	249.00	219.00	230.55	261.35	235.00	241.10	277.70	253.00	253.00	293.90
60–69	212.00	215.10	242.25	224.00	226.55	251.35	235.00	238.10	267.45	247.00	248.10	287.80
70–85	207.00	209.20	256.35	214.00	219.50	264.75	223.00	229.30	278.30	237.00	241.65	293.20
	8.5			8.6			8.7			8.8		
	p1	p5	p95	p1	p5	p95	p1	p5	p95	p1	p5	p95
18–29	259.00	266.55	304.25	267.00	272.75	314.35	271.00	285.95	331.45	277.00	293.95	349.25
30–39	251.00	255.30	313.70	255.00	258.75	322.45	259.00	265.95	332.30	273.00	282.20	338.70
40–49	259.00	263.10	305.80	263.00	265.00	318.45	265.00	270.55	328.60	265.00	277.10	344.45
50–59	261.00	261.55	308.90	262.00	262.00	316.80	265.00	275.40	334.00	280.00	284.65	349.45
60–69	254.00	258.00	294.90	257.00	258.55	301.90	258.00	264.55	320.25	260.00	274.60	343.15

70–85	245.00	251.55	302.65	253.00	257.20	313.75	262.00	269.50	332.65	265.00	275.10	340.35
